# Spiro-Flavonoids in Nature: A Critical Review of Structural Diversity and Bioactivity

**DOI:** 10.3390/molecules28145420

**Published:** 2023-07-14

**Authors:** Łukasz Pecio, Solomiia Pecio, Tomasz Mroczek, Wiesław Oleszek

**Affiliations:** 1 Department of Biochemistry and Crop Quality, Institute of Soil Science and Plant Cultivation—State Research Institute, 8 Czartoryskich Street, 24-100 Puławy, Poland; lpecio@iung.pulawy.pl (Ł.P.); wieslaw.oleszek@iung.pulawy.pl (W.O.); 2 Department of Chemistry of Natural Products, Medical University of Lublin, 1 Chodźki Street, 20-093 Lublin, Poland; tmroczek@pharmacognosy.org

**Keywords:** spiro-flavonoids, spiro-biflavonoids, spiro-triflavonoids, spiro-tetraflavonoids, spiro-flavostilbenoids, scillascillin-type homoisoflavonoids, stereochemistry, biosynthesis, biological activity, isolation

## Abstract

Based on the literature data from 1973 to 2022, this work summarizes reports on spiro-flavonoids with a spiro-carbon at the center of their structure and how this affects their isolation methods, stereochemistry, and biological activity. The review collects 65 unique structures, including spiro-biflavonoids, spiro-triflavonoids, spiro-tetraflavonoids, spiro-flavostilbenoids, and scillascillin-type homoisoflavonoids. Scillascillin-type homoisoflavonoids comprise spiro[bicyclo[4.2.0]octane-7,3′-chromane]-1(6),2,4-trien-4′-one, while the other spiro-flavonoids contain either 2*H*,2′*H*-3,3′-spirobi[benzofuran]-2-one or 2′*H*,3*H*-2,3′-spirobi[benzofuran]-3-one in the core of their structures. Spiro-flavonoids have been described in more than 40 species of eight families, including Asparagaceae, Cistaceae, Cupressaceae, Fabaceae, Pentaphylacaceae, Pinaceae, Thymelaeaceae, and Vitaceae. The possible biosynthetic pathways for each group of spiro-flavonoids are summarized in detail. Anti-inflammatory and anticancer activities are the most important biological activities of spiro-flavonoids, both in vitro and in vivo. Our work identifies the most promising natural sources, the existing challenges in assigning the stereochemistry of these compounds, and future research perspectives.

## 1. Introduction

A spiro compound, or spirane (from Latin spīra, meaning twist or coil), is an organic compound containing two or more rings connected by only one common carbon atom and exhibiting a twisted structure [1,2]. Spiro-containing substances have central or axial chirality and 3D structural properties in relation to their intrinsic rigidity [3]. These scaffolds have been found to possess pharmacological potencies and a wide range of biological activities. Spiro compounds are of increasing interest in medicinal chemistry and contribute to a large number of approved drugs and drug candidates [4].

**Figure 1:**
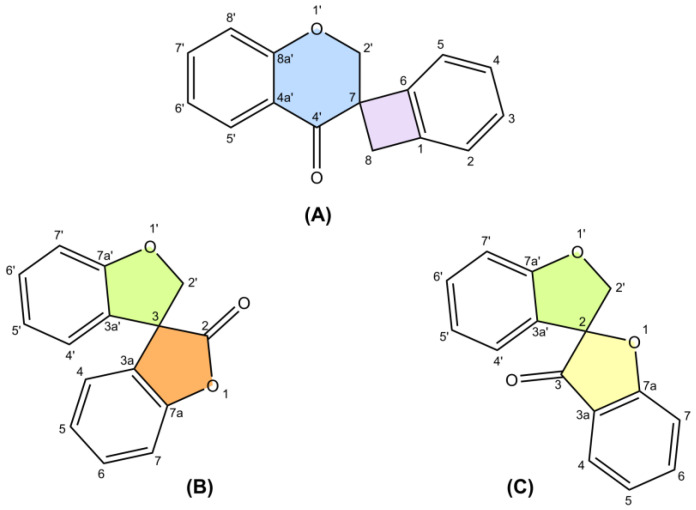
Core structures of monomeric scillascillin-type homoisoflavonoids (**A**) and oligomeric spiro-flavonoids (**B**,**C**).

Flavonoids have a 15-carbon backbone consisting of two benzene rings (rings A and B) connected by a 3-carbon linker (represented as C_6_-C_3_-C_6_ compounds). This linker can form a heterocyclic C (pyran or pyrone) ring. In the absence of this additional system, such flavonoids are called chalcones and are usually precursors in the biosynthesis of various classes of flavonoids. Spiro-flavonoids, on the other hand, are characterized by the presence of one or more spiro-carbons derived from the C-3 carbon of the corresponding flavonoid. This class of compounds is divided into two main groups: monomers and heterooligomers. Monomeric spiro-flavonoids include only scillascillin-type homoisoflavonoids with the structure spiro[bicyclo[4.2.0]octane-7,3′-chromane]-1(6),2,4-trien-4′-one (Figure 1A), which are derivatives of 3-benzylchroman-4-one [5]. The name homoisoflavonoids, which is used for compounds with a 3-benzylchroman-4-one backbone, suggests that they are formed biosynthetically in nature similar to isoflavonoids, that is, by a characteristic 2,3-aryl migration step, while they are formed by modifying the typical C_6_-C_3_-C_6_ backbone of flavonoids by inserting an additional carbon atom. Depending on the number of flavonoid units, oligomeric spiro-flavonoids are further divided into the following subclasses: spiro-biflavonoids (flavanone-flavan-3-ol or flavan-3-ol-chalcone dimers), spiro-triflavonoids (flavanone-flavan-3-ol trimers), spiro-tetraflavonoids (mixed combinations of flavan/flavan-3-ol-chalcone), and atypical spiro-flavostilbenoids (dimers and flavanone-stilbenoid trimers) [6,7]. The presence of the 2*H*,2′*H*-3,3′-spirobi[benzofuran]-2-one (Figure 1B) or 2′*H*,3*H*-2,3′-spirobi[benzofuran]-3-one (Figure 1C) systems connects all compounds belonging to the class of oligomeric spiro-flavonoids. To date, no spiro-flavonoids have been described that have an iso- or neoflavonoid unit in their structure.

The only review paper on spiro-flavonoids was published by Fedorova et al. [8] and covered eight spiro-biflavonoids and one spiro-triflavonoid that had been isolated prior to 2010, without any discussion of their stereochemistry. Thanks to modern and increasingly available spectroscopic techniques, more than 50 additional spiro-flavonoids have been characterized to date, resulting in a total of 65 unique structures, and are reported here. Difficulties in assigning stereochemistry, artifacts, and discovering spiro-flavonoids already described under another name were discussed.

This review summarizes the literature data for the years 1973–2022, including the authors’ studies, on the occurrence in nature, methods of isolation, elucidation of structures, assignment of stereochemistry, bioactivity, and putative biosynthesis of spiro-flavonoids.

POWO “Plants of the World Online” [9] and IPNI “International Plant Names Index” [10] were used to verify the plant and family names (Table 1). Common numbering of carbon atoms of spiro compounds was used according to Nakashima et al. (2016) and Pecio et al. (2023) [7,11].

## 2. Occurrence in Nature

Spiro-flavonoids are a fairly large group of polyphenolic compounds (although less numerous by an order of magnitude than biflavonoids [12]), and to our knowledge a total of 65 have been described, including spiro-biflavonoids, spiro-triflavonoids, spiro-tetraflavonoids, spiro-flavostilbenoids, and scillascillin-type homoisoflavonoids (Table 2, Table 3, Table 4, Table 5 and Table 6). These first four groups of compounds reported here are composed of two or more nonidentical flavonoid and/or stilbene units, whereas homoisoflavonoids, with one exception, are monomeric structures. Interestingly, to date, no glycosylated forms of spiro-flavonoids have been described. Spiro-biflavonoids are the most abundant group, followed by scillascillin-type homoisoflavonoids and spiro-flavostilbenoids (29, 17, and 13 compounds described, respectively), while only four spiro-tetraflavonoids and two spiro-triflavonoids were described. They have been isolated from eight plant families represented by 46 species. The main sources of spiro-flavonoids are plants belonging to the Asparagaceae (34 compounds), Pinaceae (16), and Thymelaeaceae (10) families, especially species from *Yucca* (18 compounds), *Larix* (10), *Abies* (9), *Drimiopsis* (8), and *Daphne* (7) genera. In the case of Asparagaceae, most of the compounds (sixteen) belong to the group of scillascillin-type homoisoflavonoids, thirteen to spiro-flavostilbenoids, and five to spiro-biflavonoids. On the other hand, Pinaceae and Thymelaeaceae were predominantly spiro-biflavonoid sources, as were Cupressaceae and Pentaphylacaceae. Both woody aerial parts and roots were used to isolate spiro-biflavonoids and spiro-flavostilbenoids. Spiro-tri and spiro-tetraflavonoids were isolated from twigs and bark, and the main sources of scillascillin-type homoisoflavonoids were bulbs.

**Table 1:** Spiro-flavonoids described between 1973 and 2022.

Family	Plant	Part	Compound	References
Asparagaceae	*Bessera elegans* Schult.f.	bulbs	**57**	[13]
	*Chionodoxa luciliae* Boiss.	bulbs	**49–52**	[14]
	*Drimiopsis barteri* Baker	bulbs and leaves	**50, 56, 60, 61, 64**	[15]
	*Drimiopsis burkei* Baker	bulbs	**53, 62**	[15]
	*Drimiopsis maculata* Lindl. & Paxton	bulbs	**52, 56**	[16,17]
	*Eucomis schijffii* Reyneke	bulbs	**49**	[17,18]
	*Furcraea bedinghausii* K.Koch	roots	**38, 39**	[19]
	*Ledebouria graminifolia* (Baker) Jessop	bulbs	**58, 61**	[20]
	*Ledebouria hyderabadensis* M.V.Ramana, Prasanna & Venu	bulbs	**49**	[21]
	*Ledebouria ovatifolia* (Baker) Jessop	bulbs	**49, 50**	[22]
	*Ledebouria socialis* (Baker) Jessop	bulbs	**63, 64**	[22]
	*Merwilla natalensis* (Planch.) Speta	bulbs	**49**	[23]
	*Muscari armeniacum* H.J.Veitch	bulbs	**52, 54**	[24]
	*Muscari botryoides* (L.) Mill.	bulbs	**52, 54, 56**	[24]
	*Muscari comosum* (L.) Mill.	bulbs	**53**	[25]
	*Muscari neglectum* Guss. ex Ten.	bulbs	**53–56**	[26]
	*Scilla scilloides* (Lindl.) Druce	bulbs	**49, 51, 52, 59**	[27]
	*Yucca gloriosa* L.	roots	**38–48**	[11,28,29]
	*Yucca schidigera* Roezl ex Ortgies	bark	**1, 5–7, 29, 36–41, 44, 46–48**	[30,31,32,33]
Cistaceae	*Fumana procumbens* (Dunal) Gren. & Godr.	whole plant	**23**	[34]
Cupressaceae	*Glyptostrobus pensilis* (D.Don) K.Koch	trunk bark	**1–4, 10, 11**	[35]
Fabaceae	*Caesalpinia sappan* L.	heartwood	**65**	[36]
Pentaphylacaceae	*Anneslea fragrans* Wall.	twigs	**2, 8, 9, 30**	[37,38]
Pinaceae	*Abies chensiensis* Tiegh.	aerial parts	**1–3**	[39]
	*Abies delavayi* Franch. var. *delavayi*	aerial parts	**1**	[40]
	*Abies georgei* Orr	aerial parts	**1, 12**	[41]
	*Abies sachalinensis* (F.Schmidt) Mast.	bark	**1, 4, 12–17**	[42]
	*Larix decidua* Mill.	bark	**1–3**	[43]
	*Larix gmelinii* (Rupr.) Kuzen.	bark	**1, 14, 18, 31**	[6,44,45]
	*Larix olgensis* Henry var. *koreana* Nakai	bark	**14, 16, 19–21**	[46]
	*Larix sibirica* Ledeb.	bark	**1, 14, 31**	[44]
	*Pinus massoniana* Lamb.	bark	**35**	[47]
	*Tsuga longibracteata* W.C.Cheng	bark	**1, 2**	[48]
Thymelaeaceae	*Daphne aurantiaca* Diels	stem bark	**22, 24**	[49]
	*Daphne feddei* H.Lév.	stem bark	**22, 24, 26, 27**	[50]
	*Daphne genkwa* Siebold & Zucc.	roots	**23**	[51]
	*Daphne kiusiana* Miq.	stem	**22, 24**	[52]
	*Daphne kiusiana* var. *atrocaulis* (Rehder) F.Maek.	stem	**22**	[53]
	*Daphne linearifolia* Hart	aerial parts	**23–25, 28**	[54]
	*Daphne mucronata* Royle	shoots	**23**	[55]
	*Daphne odora* Thunb.	roots	**22, 24**	[56,57]
	*Edgeworthia chrysantha* Lindl.	stem and twigs	**22, 24, 32–34**	[58]
	*Stellera chamaejasme* L.	roots	**23**	[59]
	*Thymelaea microphylla* Coss. & Durieu	roots	**23**	[60]
	*Wikstroemia indica* (L.) C.A.Mey.	roots	**22, 23**	[61,62]
Vitaceae	*Vitis amurensis* Rupr.	seeds	**16**	[63]

## 3. Methods of Extraction and Isolation

Due to their differing lipophilicity, spiro-flavonoids were extracted using different organic solvents and their mixtures, mainly with water (Table 7). Therefore, spiro-biflavonoids were extracted most frequently with EtOH, MeOH, and their aqueous solutions (in more than ^2^/_3_ of the publications); spiro-triflavonoids with ethyl acetate (EtOAc) or an aqueous solution of EtOH; spiro-tetraflavonoids with an aqueous solution of acetone; and spiro-flavostilbenoids with MeOH and its aqueous solutions or acetone. On the other hand, scillascillin-type homoisoflavonoids were extracted from plant material using dichloromethane (DCM), its solution with MeOH, or MeOH alone, but also diethyl ether. In general, MeOH, EtOH, and their aqueous solutions were the extractants most commonly used. The extraction process was usually carried out by maceration at room temperature (28 papers), 15 articles reported extraction at boiling point (heat reflux method), and the remaining 13 publications did not provide this information. Column chromatography (CC) and liquid–liquid extraction technique (LLE) were frequently used as the first step of the compound isolation procedure, and the LLE fraction obtained with EtOAc was used in further steps. The pre-purification of crude extracts by CC was most commonly performed by normal phase, followed by reversed phase, and much less frequently by gel filtration chromatography (GPC). Normal-phase CC mainly used gradients of many different solvents: CHCl_3_, DCM, diethyl ether, EtOAc, *n*-hexane, and MeOH. Reversed-phase CC almost exclusively used gradients of MeOH and H_2_O, while MeOH was used for CC GPC. Interestingly, in the first stage of spiro-biflavonoid isolation, LLE was used more extensively than CC, while the opposite was true for scillascillin-type homoisoflavonoids. The next isolation step involved the use of CC (mainly normal phase), regardless of the spiro-compound group, followed by the use of thin-layer chromatography (TLC), medium-pressure-, and high-performance liquid chromatography (MPLC and HPLC). The final isolation steps usually involved CC combined with MPLC, HPLC, and TLC.

## 4. Stereochemistry of the Isolated Spiro-Flavonoids

The presence of up to 8 stereogenic centers in the known spiro-flavonoid molecules and the associated possibility of up to 128 pairs of enantiomers (2^8^ = 256) occurring in nature makes it a challenging task to assign the relative configuration (RelC), let alone the absolute configuration (AbsC). When molecules with multiple stereogenic centers are studied, the determination of the relative configuration is particularly important for the assignment of the AbsC. In such a case, the determination of the AbsC by a single chiroptical spectroscopic method may lead to an incorrect assignment of the absolute configuration, especially if no prediction is made by quantum mechanical (QM) calculation methods and only by comparison with the spectra of previously described compounds (empirical methods). This is because in some cases, even QM (e.g., ECD)-calculated spectra may match more than one diastereoisomer, and interactions of different chromophores should be taken into account for correct AbsC assignment [64,65].

Spiro-flavonoids are compounds that contain four-, five-, and six-membered rings in their structure. To determine the relative configuration in cyclic compounds with three- to six-membered rings, which exhibit a predictable conformational behavior, it is usually sufficient to extract some NMR parameters, such as the ^1^H-^1^H vicinal coupling constants (^3^*J*_HH_) (and the corresponding dihedral angles via the Karplus equation [66]) and nuclear Overhauser effect spectroscopy (NOESY) correlations. Additional information can be obtained in systems containing electronegative substituents directly attached to the α-carbon (i.e., oxygen, nitrogen, or halogen) can be obtained by measuring the values of the heteronuclear coupling constants—if the value of ^2^*J*_HC_ is large (4–7 Hz), the position of the electronegative substituent is gauche relative to the geminal proton, and becomes small (0–2 Hz) if it is antiperiplanar. On the other hand, ^3^*J*_HC_, similar to ^3^*J*_HH_, follows the Karplus relation and can also be used to determine additional constraints on dihedral angles—the values of this constant are lower (1–3 Hz) for gauche than for anti-periplanar conformation (5–8 Hz) [31,67,68,69]. An empirical rule developed by Nakashima et al. (2016) [11], which allows easy determination of the relative configuration for the spiro-carbon (C-3) and its neighboring carbon (C-2), was applied on the basis of the observation of ^13^C NMR chemical shifts for some spiro-flavonoids (abiesinols A-H, yuccalides A-C, yuccaols A-E, and gloriosaols A-E) reported in this review. Namely, for the ^13^C NMR shifts measured in the acetone-*d_6_* and methanol-*d_4_*, the γ-lactone carbonyl group (C-4) located in the δ_C_ range of 174.6–176.9 indicates a *syn*-relationship between C-4 and the aromatic substituent located at the C-2 carbon, due to the anisotropic shielding effect exerted by the aromatic substituent on C-4, and thus the *rel*-(2*R*,3*S*) configuration. On the other hand, the γ-lactone carbonyl group, which has an *anti*-relationship with the aromatic substituent in C-2, has chemical shifts in the range δ_C_ 178.6–181.1, indicating a *rel*-(2*R*,3*R*) configuration. An additional tool, increasingly used for RelC determination, is the use of quantum-based Gauge-Independent Atomic Orbital (GIAO) NMR shift calculations via a modified DP4+ probability method, which allows one to determine with high probability which of the possible stereoisomers is the correct one [70].

The techniques used to determine AbsC are direct methods, such as electronic and vibrational circular dichroism (ECD and VCD), but also indirect (or relative) methods, such as circular dichroism using empirical methods, NMR with chiral derivatization agents [71] (e.g., Mosher’s method [72,73]). The crux of Mosher’s method lies in the difference in anisotropic effects between two diastereomers prepared from the substrate (molecule) of interest with a pair of enantiomeric chiral derivatizing agents (CDAs). In practice, the differences in chemical shifts (Δδ^RS^ or Δδ^SR^) between the two substrate derivatives are used to assign AbsC. The most common CDA is MTPA (α-methoxy-α-trifluoromethylphenylacetic acid), commonly known as Mosher’s reagent. This method typically involves the double derivatization of substrates with two CDA enantiomers and therefore requires a relatively large sample amount. In recent years, however, ECD has become a particularly popular method for determining AbsC. ECD involves an electron transition and can therefore be predicted theoretically by QM calculations. With the measured CD spectrum with the TDDFT (Time-Dependent Density-Functional Theory)-calculated CD spectrum of an assumed configuration, one can easily assign the AbsC of a chiral compound. Another advantage of this technique is that only sub μg amounts of the sample needed to obtain the spectrum, which measures the difference in the chiral response of a molecule to UV/Vis modulation between the left and right polarized states [74]. To our knowledge, X-ray diffraction (XRD), which is a direct method that can be used to determine AbsC and is considered to be very reliable [75], has only been used for the determination of RelC of spiro-flavonoids.

### 4.1. Spiro-Biflavonoids

#### 4.1.1. Larixinol Sub-Group (**1**–**21**) and Yuccaone A (**29**)

One of the first spiro-biflavonoids discovered was larixinol (**1**), isolated from the bark of *Larix gmellini* (Rupr.) Kuzen. [45,76]. Its structure (Table 2) and its relative configuration were assigned by X-ray crystallographic analysis as *rel*-(2*R*,3*R*,2′*R*,3′*R)*, but its absolute configuration was not confirmed until almost a quarter of a century later by Wada et al. (2009), who isolated abiesinol E (=larixinol) from the bark of *Abies sachalinensis* (F.Schmidt) Mast., and assigned its absolute configuration as 2*R*,3*R*,2′*R*,3′*R* using Mosher’s method [42]. A total of eight abiesinols (A–H) (**1**, **4**, **12**–**17**) were reported in the same article. Their relative configurations were determined using ^3^*J*_HH_ coupling constants and NOEs, and their AbsCs were determined using the Mosher method and by comparing optical rotations and ECD spectral data. Interestingly, abiesinol G (**16**) had the same chemical structure and stereochemistry as vitisinol first isolated from the seeds of *Vitis amurensis* Rupr. [63], while abiesinol C = olgiensisinol A (14) first isolated from stem bark of *Larix olgensis* Henry var*. koreana* Nakai [46] (Table 2). In the latter work, the authors used spatial anisotropic shielding effects for the γ-lactone carbon and NOESY to determine the RelC, and a comparison of ECD spectra with the literature data was used to determine the AbsC of olgensisinol A and B (**19**). The authors of this work also reported the relative configuration of other unusual spiro-biflavonoids, olgiensisinols C and D (**20**, **21**), but no attempt was made to determine their AbsC. Yuccalechins A-C (**5**–**7**) (Table 2), extracted from the bark of *Yucca schidigera* and described by Pecio et al. (2019) [31], are another example of compounds whose AbsC was determined with high reliability using measurements of ECD spectra and TDDFT of calculated ECD spectra. In this publication, the authors used measurements of different coupling constants (^3^*J*_HH_, ^1^*J*_HC_, ^2^*J*_HC_, and ^3^*J*_HC_), spatial anisotropic shielding effects for ^13^C NMR chemical shifts, and NOESY correlations for the determination of RelC, as well as QM calculations with the DP4+ method. Similarly, spatial anisotropic shielding effects of ^13^C NMR and NOESY correlations were used to determine RelC, and QM calculations of ECD spectra were used to determine AbsC of fragranols B and C (**8**, **9**) (Table 2) by Omar et al. (2020) [38].

Several papers have also been published in which RelC/AbsC have been determined in a way that casts doubt on their accuracy. For example, Li et al. (2009) [39] report 3-*epi*-larixinol (**2**) and 3,2′-*epi*-larixinol (**3**) whose AbsC were implicitly suggested using ^3^*J*_HH_ coupling constants, NOESY correlations, and optical rotations. Unfortunately, the authors did not measure ECD spectra and nor did they use Mosher’s method, which could be critical to determining the stereochemistry. Following this line of thought, it turns out that if the absolute configuration of 3,2′-*epi*-larixinol, i.e., (2*S*,3*R*,2*R*,3*S*), is taken as true, it corresponds exactly to yuccalechin B (**6**), but its optical rotations differ in sign and value (+25.0 vs. −175.0, respectively), and the chemical shifts of ^1^H and ^13^C differ significantly, e.g., for H-2/C-2, C-4, and H-2/C-2′ (δ_H_/δ_C_ 5.79/95.3, 181.0, 4.57/83.1 vs. 6.13/90.6, 177.1, 4.99/81.6). Another example that raises questions about the use of empirical methods to determine AbsC is the work of Xiong et al. (2020) [35], which describes spiropensilisol A and B (**10**, **11**). The relative configurations of these compounds were determined using ^3^*J*_HH_ coupling constants, ^13^C NMR anisotropic shielding effects, and NOEs. Using this method, the authors proposed the AbsC of spiropensilisol B as (2*S*,3*S*,2′*R*,3′*S*)-**11**, which coincides with the configuration of yuccalechin C (**7**). However, the ECD spectra show a significant difference around 220 nm between these two compounds. On the other hand, based only on ^1^H and ^13^C NMR chemical shifts and optical rotations, Yang et al. (2011) [41] proposed 13-hydroxylarixinol as a new natural compound isolated from *Abies georgei* Orr. Assuming that the AbsC determination for this compound by this route is correct, its absolute configuration corresponds to that of abiesinol A (**12**), which questions the novelty of this discovery. Similarly, in the work of Piacente et al. (2004) [33], the authors reported the presence of larixinol only on the basis of ^1^H and ^13^C NMR values, while Pecio et al. (2019) [31] did not confirm the presence of larixinol, but reported three compounds in the same plant with the same planar structure but different stereochemistry, i.e., yuccalechins A-C. The presence of an unusual spiro-biflavonoid, yuccaone A (**29**), was also reported in the bark of *Y. schidigera* [32,33], the RelC of which was determined by NOESY correlation as (2R*,4R*,2′S*,3′R*). On the other hand, Fedorova et al. (2007) [6] only partially assigned the RelC of spiro-biflavonoid larisinol (**18**) using ^1^H/^13^C NMR analysis.

#### 4.1.2. Daphnodorin C Sub-Group (**22**–**28**)

Daphnodorin C (**22**) is the simplest representative of a group of compounds isolated from a *Daphne* Tourn. ex L. genus, with a chemical structure quite similar to the larixinol group (Table 2). However, instead of a γ-butyrolactone ring, it has a 3-oxotetrahydrofuran ring, indicating a different biosynthetic pathway. This compound, first described by Baba et al. in 1986, was isolated from the roots and bark of *Daphne odora* Thunb. [77]. Its relative configuration was determined by XRD and NOESY correlations, and AbsC (2*S*,3*S*,2′*S*) was assigned by chemical degradation (H_2_SO_4_/EtOH), resulting in a product with a known absolute configuration (daphnodorin A) [78]. Another representative of this group of compounds is genkwanol A (**23**), isolated from the roots of *Daphne genkwa* Siebold & Zucc. [51]. Its structure and AbsC (2*R*,3*R*,2′*R*,3′*S*) were determined by Baba et al., 1987, by chemical transformation (HCl/MeOH) to daphnodorin B and comparison of Cotton effects observed in the ECD spectra [78]. AbsC (2S,3S,2′R,3′S) of daphnodorin I (**24**), which is a diastereoisomer of genkwanol A, was determined in a similar manner, i.e., by chemical conversion of **24** to daphnodorin B (using HCl/MeOH), and comparison of their ECD spectra [57]. Malafronte et al. (2012) isolated and elucidated the structures of 4′-methylgenkwanol A (**25**) and 2″-hydroxygenkwanol A (**28**) from *Daphne linearifolia* Hart. The compounds had identical AbsC, (2*R*,3*S*,2′*R*,3′*S*), determined by comparison of ^1^H/^13^C and ECD data with those of genkwanol A [54]. 2″-Methoxy-daphnodorin C (**26**) and 2″-methoxy-2-*epi*-daphnodorin C (**27**) were isolated from *Daphne feddei* H.Lév. and their stereochemistry was determined by NMR data consistent with daphnodorin C and XRD analysis, which confirmed their RelC as (2R*,3S*,2′R*)-**26** and (2R*,3S*,2′S*)-**27**, respectively [50].

### 4.2. Spiro-Triflavonoids (***30***–***31***)

Fragranol A, a spiro-triflavonoid containing two spiro-centers and six chiral carbon atoms, was isolated from twigs of *Anneslea fragrans* (Table 3) [37]. The RelC of the comp. **30** was determined by NOESY correlations and spatial anisotropic shielding effects around the spiro-centers. Fragranol A AbsC was confirmed by QM ECD calculations and assigned as (2*S*,3*S*,2′*S*,3′*R*,2″*R*,3″*S*). Ivanova et al. (2006) partially assigned the RelC of spiro-triflavonoid triflarixinol (**31**) using ^1^H/^13^C NMR analysis, infrared spectroscopy, and melting point measurements of recrystallized compounds [44]. However, the authors did not attempt to determine the AbsC.

### 4.3. Spiro-Tetraflavonoids (***32***–***35***)

Edgechrins (Table 4) are condensed daphnodorin dimers—daphnodorin C-(4′β→6‴)-daphnodorin A, daphnodorin C-(4′β→6‴)-daphnodorin B, daphnodorin A-(4‴β→8)-daphnodorin C (comp. **32–34**, respectively), and their structures were elucidated by the ^1^H-, ^13^C- and DEPT, COSY, and NOESY NMR spectra [58]. The HMBC correlations were used to determine the linkage between dimers. Their AbsC were assigned by biogenetic considerations and supported by the empirical method: comparison of Cotton effects observed in the ECD spectra. The ECD spectra were compared with daphnodorins A, B, and C. The authors report that they performed NOESY NMR measurements. However, they do not present the use of NOE to determine RelC or to confirm AbsC against known biogenetic monomers. Furthermore, the absolute configurations of the structures are not explicitly reported. Moreover, the absolute configurations in the text of the article did not match the stereochemistry of the structures shown in the figures, which seems to undermine the correctness of the stereochemistry determination. Zhou et al. (2020) [47] reported another interesting tetrameric spiro-flavonoid, pinuspirotetrin (**35**), which they described as a proanthocyanidin. It was isolated from the bark of *Pinus massoniana* Lamb. This compound represents the first heterodimeric (4 → 8 linked) proanthocyanidin containing a spiro-type and an A-type dimer. The authors determined the AbsC of this unusual structure in a plausible manner, using combined spectroscopic methods, i.e., NOESY NMR correlations and an empirical method based on the use of ECD spectra and Cotton effects for known compounds, to establish the stereochemistry of **35**.

### 4.4. Spiro-Flavostilbenoids (***36***–***48***)

Spiro-flavostilbenoids are derivatives of *trans*-resveratrol (*trans*-3,4′,5-trihydroxystilbene) or *trans*-3,3′,5,5′-tetrahydroxy-4′-methoxystilbene (THMS) and naringenin. Structures of yuccaols A-E (**36**–**40**) were first described by Oleszek et al. (2001) [30] and Piacente et al. (2004) [33], while yuccalides A-C (**41**–**43**) and gloriosaols A-E (**44**–**48**) (Table 5) were described throughout the next decade [11,28,29]. The authors of these articles reported only the relative configurations for the isolated compounds—*rel*-(2*R*,3*S*) for compounds **36**, **39**, **41**, **43**, *rel*-(2*R*,3*R*) for compounds **37**, **38**, **40**, **42**, *rel*-(2*R*,3*R*,2′*R*,3′*R*) for **44**, *rel*-(2*S*,3*S*,2′*R*,3′*R*) for **45**, *rel*-(2R,3*S*,2′*R*,3′S) for **46**, *rel*-(2*R*,3*R*,2′*R*,3′*S*) for **47**, and *rel*-(2*R*,3*S*,2′*R*,3′*R*) for **48**. These were determined by NOESY correlations and, in the case of gloriosols A-E, by QM geometry calculations and GIAO ^1^H chemical shifts. Chiroptical properties in the form of optical rotations have been measured for all members of the group, except for gloriosaols D and E isolated as a mixture. However, ECD spectra have been obtained for yuccalides A-C and yuccaols C-E. More recently, Pecio et al. (2023) [7] established AbsCs for yuccaols A-E, yuccalide A, and gloriosaols A and C-E extracted from the bark of *Y. schidigera* by comparing experimental and calculated TDDFT ECD spectra. The absolute configurations of yuccalides B-C and gloriosaol B have not yet been determined.

### 4.5. Scillascillin-Type Homoisoflavonoids (***49***–***65***)

The first homoisoflavonoids with a spiro-structure, scillascillin (**49**) and 2-hydroxy-7-*O*-methyl-scillascillin (**50**), were isolated from the bulbs of *Scilla scilloides* (Lindl.) Druce by Kouno et al. (1973) (Table 6) [5]. Corsaro et al. (1992) measured Cotton effects for compounds **49** and **51** (2-hydroxy-scillascillin) and assigned their AbsCs as 3*R* [14]. Adinolfi et al. (1990) determined the AbsC as 3*R* of the scillascillin-type homoisoflavonoids of *Muscari* spp., i.e., isomuscomosin (**52**), muscomosin (**53**), 3′,4′,5-trihydroxy-7-methoxyspiro[2*H*-1-benzopyran-3(4*H*),7′-bicyclo[4.2.0]octa[1,3,5]trien]-4-one (**54**), 5,5′-dihydroxy-4′,7-dimethoxyspiro[2*H*-1-benzopyran-3(4*H*),7′-bicyclo[4.2.0]octa[1,3,5]trien]-4-one (**55**), and 3′,5-dihydroxy-4′,7-dimethoxyspiro[2*H*-1-benzopyran-3(4*H*),7′-bicyclo[4.2.0]octa[1,3,5]trien]-4-one (**56**) using several methods [79]. The compounds were first methylated with diazomethane in ether, and **52**–**54**, **56** gave the same dextrorotatory product, namely 5-hydroxy-3′,4′,7-trimethoxyspiro[2*H*-1-benzopyran-3(4*H*),7′-bicyclo[4.2.0]octa[1,3,5]trien]-4-one. This product was then esterified at C-5 with *p*-bromobenzoyl chloride, followed by reduction with sodium borohydride to give a deoxygenated compound at C-4, which was again *p*-bromobenzoylated. The RelC and AbsC of the products were determined by NMR and confirmed by measurements of their CD curves and XRD [79]. The AbsC of **55** was assigned based on the measurement of its Cotton effects and X-ray crystallography. Waller et al. (2013) could not directly determine AbsC at C-2 of 2-hydroxy-7-*O*-methyl-scillascillin (**50**) due to the instability of the hemiacetal ring [22]. Acetylation of this compound solved this problem by yielding two diastereomers, and their AbsCs were confirmed as 2*R*,3*R* and 2*S*,3*R* using NMR and ECD experiments by comparing experimental and calculated TDDFT ECD spectra. The AbsC of comp. **57** (5,7,3′-trihydroxy-4′-methoxy-6-methylspiro[2*H*-1-benzopyran-3(4*H*),7′-bicyclo[4.2.0]octa[1,3,5]-trien]-4-one) was determined to be 3*R* by extensive NMR analysis, based on the similarity of the experimental CD spectra to those of comp. **59** (scillavone A) [27]. In contrast, Nishida et al. (2008) determined the RelC of comp. **59** by XRD and then its AbsC (3*R*) by comparison of Cotton effects with the literature data [79]. The AbsC of compounds **58** and **60**–**64** has not yet been determined [15,20,22], although the literature to date indicates that the scillascillin-type homoisoflavonoids described so far have only the 3*R* configuration.

Compound **65** (protosappanin D) belongs to the group of dimeric protosappanin-type homoisoflavonoids, which are characterized by the presence of a biphenyl sappanin moiety forming a methyloxocane ring. It has a very interesting structure with two spiro systems, but its spectral and physicochemical properties have not been reported, not to mention its AbsC. Washiyama et al. (2009) only mentioned that it was extracted from Sappan Lignum (*Caesalpinia sappan* heartwood) purchased from Uchida Wakanyaku Ltd. [36].

## 5. Biosynthesis of Spiro-Flavonoids

Since studies on the biosynthesis of spiro-flavonoids have not yet been performed, it seems appropriate to present possible mechanisms in light of the knowledge of flavonoid biosynthesis. It is generally accepted that flavonoids are synthesized in the cytosol, and the enzymes involved are connected to the endoplasmic reticulum [80,81]. The pathway begins with the formation of the C_6_-C_3_-C_6_ skeleton by chalcone synthase (CHS), which catalyzes the synthesis of naringenin chalcone from one molecule of *p*-coumaroyl-CoA and three molecules of malonyl-CoA. In the biosynthesis of flavonoids, the unstable chalcone is stereospecifically converted to the flavanone, (2*S*)-naringenin, by chalcone isomerase (CHI). In the absence of CHI, the isomerization of chalcone occurs spontaneously, producing the racemic mixture of (2*S*)-naringenin and (2*R*)-naringenin, but the enzyme-catalyzed reaction is 107 times faster than the spontaneous one [82,83]. (2*S*)-Flavanones are the exclusive substrates of downstream enzymes of the flavonoid pathway, and thus CHI guarantees the efficient formation of biologically active (2*S*)-flavonoid isomers. Interestingly, *Arabidopsis* mutants lacking CHI activity have been reported to accumulate only trace amounts of flavonoids [84].

During flavonoid biosythesis, flavanones can undergo desaturation by flavone synthase I, which produces apigenin from (2*S*)-naringenin. Subsequently, flavanones can also be regioselectively and stereoselectively converted to (2*R*,3*R*)-dihydroflavonols (the presence of the 3-hydroxyl group reverses the priority sequence of the Cahn–Ingold–Prelog specification of absolute configuration [85]) via the intermediate C-3 flavanone radical [86] by hydroxylation of a pro-*R* hydrogen in C-3 catalyzed by both flavanone 3β-hydroxylase and flavone synthase [87]. These enzymes are classified as soluble 2-oxoglutarate-dependent dioxygenases due to their requirement for the cofactors 2-oxoglutarate, molecular oxygen, ferric iron (Fe^2+^), and ascorbate. Only (2*S*)-naringenin, but not the (2*R*)-enantiomer, was reported to be a substrate for flavanone 3β-hydroxylase from *Petunia hybrida* [88]. On the other hand, in addition to the oxidation of *trans*-(2*R*,3*R*)-dihydrokaempferol, but not that of *trans*-(2*S*,3*S)*-dihydrokaempferol to kaempferol, flavonol synthase catalyzes the conversion of (2*S*)- or (2*R*)-naringenin to the corresponding *trans*-dihydrokaempferol [87]. Subsequently, dihydroflavonols are reduced to flavan-3,4-diols by dihydroflavonol 4-reductase and leucoanthocyanidin reductase during the biosynthesis of flavan-3-ols, also involving anthocyanidin reductase and anthocyanidin synthase.

Stilbene synthase is closely related to CHS, catalyzing a similar set of reactions, but performing a divergent cyclization reaction to generate stilbenes with the C_6_-C_2_-C_6_ backbone, which compete with CHS for the same substrates (*p*-coumaroyl-CoA and malonyl-CoA) [89]. There is also evidence that stilbene synthase (STS) has evolved from CHS several times during evolution [81]. Both chalcone and stilbene synthase lead to a tetraketide intermediate in the form of CoA thioester followed by C_6_-C_1_ Claisen condensation to give chalcone in the case of CHS, while C_2_-C_7_ aldol condensation with loss of CO_2_ leads to resveratrol in the case of STS. Competition between two metabolic pathways, the flavonoid pathway controlled by chalcone synthase on the one hand and the stilbene pathway controlled by STS on the other, may occur with respect to precursor availability. Deprivation of CHS of its substrates results in reduced levels of some flavonoids, such as rutin or naringenin in transgenic tomato, levels of flavonols in recombinant strawberry, or pale fawn color in *Arabidopsis* [90,91,92,93]. The expression of stilbene synthase genes has been shown to contribute to the constitutive defense against pathogens in grapevines [94]. It has been suggested that the formation of resveratrol dimers and higher oligomers, thought to be phytoalexins, is a plant response to stress caused by fungal infection, UV radiation, or physical trauma [95,96,97]. Various modifications can occur on the stilbene core under the catalytic action of peroxidases; this is the basis of the polymerization process leading to the formation of oligomeric structures of resveratrol, as well as other reactions involving “decorating” enzymes (methyltransferases, prenyltransferases, hydroxylases, and glucosyltransferases). This oxidative coupling of resveratrol to form oligomeric compounds was extensively studied in *Vitis vinifera* L. [98] and proceeds through the coupling of oxidatively generated phenoxyl radicals, as originally proposed [99]. The dimerization process can proceed in several regioisomeric coupling modes (3-8′, 8-10′, 8-8′, and 8-12′). After this step, the formed highly reactive *p*-quinone methides can undergo a series of regioselective Friedl–Crafts reactions, nucleophilic trappings, or tatutomerizations, leading to an impressive number of possible products: resveratrol derivatives. However, the presence of oligomeric forms of resveratrol has not been reported in plants containing spiro-flavonoids that contain a stilbene moiety in the structure.

Existing hypotheses regarding the mechanisms of spiro-flavonoid formation require at least two oxidation steps and suggest that these compounds are formed by trapping a putative carbocation intermediate in the oxidative conversion of flavanone to flavonol [30,45], or by radical coupling of two oxidatively generated phenoxyl radicals [7,47]. The hypothesis that assumes the formation of a flat carbocation does not specify how exactly it would be formed. However, the free radical mechanism also produces a flat trigonal radical that can react on either side. In general, the process of formation of spiro-flavonoids may not be directly related to the biosynthesis in the cytoplasm. Their formation outside photosynthetic tissue could be due, as in the case of resveratrol oligomers, to interference with ion transport and related redox processes [100]. Rather, the necessary substrates can be transported across cell membranes to the lignifying zone in the cell wall, where they undergo enzymatic oxidation (dehydrogenation). As in lignin synthesis by radical coupling of monolignols, the enzymes laccase and peroxidase may be involved in this oxidation, but further experimental data should be collected to support these statements [101,102]. Interestingly, Pecio et al. (2023) [7] observed that all spiro-flavonoids isolated from the bark of *Y. schidigera* and the spiro-biflavonoid fragranol B (**8**), as well as the spiro-triflavonoid fragranol A (**30**) from the bark of *Anneslea fragrans*, have (2*R*)-naringenin in their structures [37,38], while abiesinoles A-H from the bark of *Abies sachalinensis* contain (2*S*)-naringenin or (2*S*)-eriodictyol [42]. This indicates a certain stereoselectivity in their formation.

**Scheme 1:**
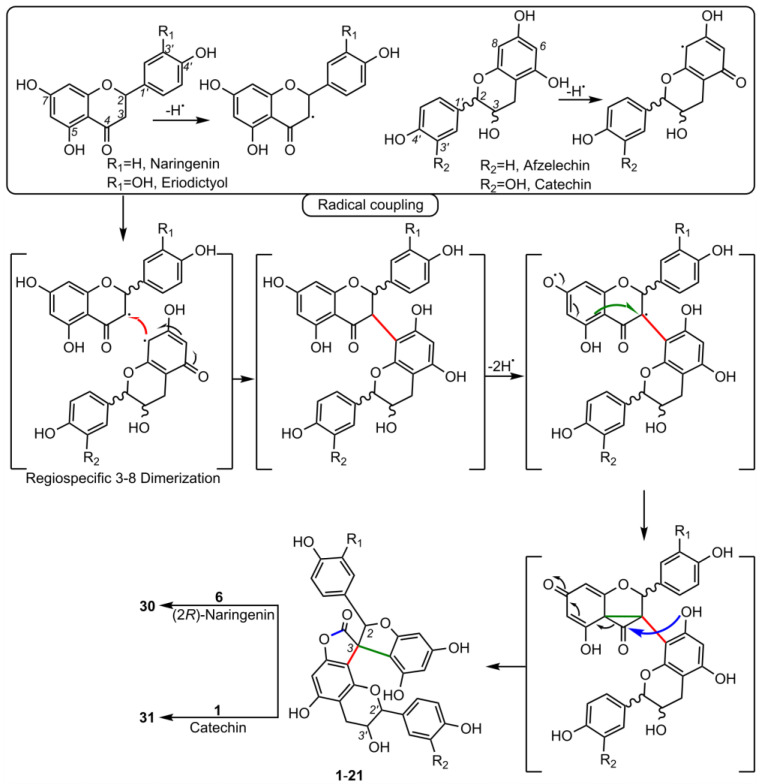
Hypothetical biosynthetic routes to larixinol-type spiro-flavonoids (adapted from Pecio et al. (2023) [7]).

Scheme 1 shows a hypothetical biosynthetic relationship between flavanones, flavan-3-ols, and larixinol-type spiro-bi- and spiro-triflavonoids (**1**–**21**, **30**–**31**, respectively) according to the mechanism proposed by Zhou et al. (2020) and Pecio et al. (2023) [7,47]. Their formation begins with the regiospecific coupling of two radicals (linkage 3–8). In the next step of oxidation, a triplet diradical is formed, which undergoes a Favorskii-type rearrangement by forming a cyclopropanone intermediate [103]. This intermediate is lactonized by nucleophilic substitution to form a benzofuranone ring through preferred *exo*-trig cyclization [104,105].

**Scheme 2:**
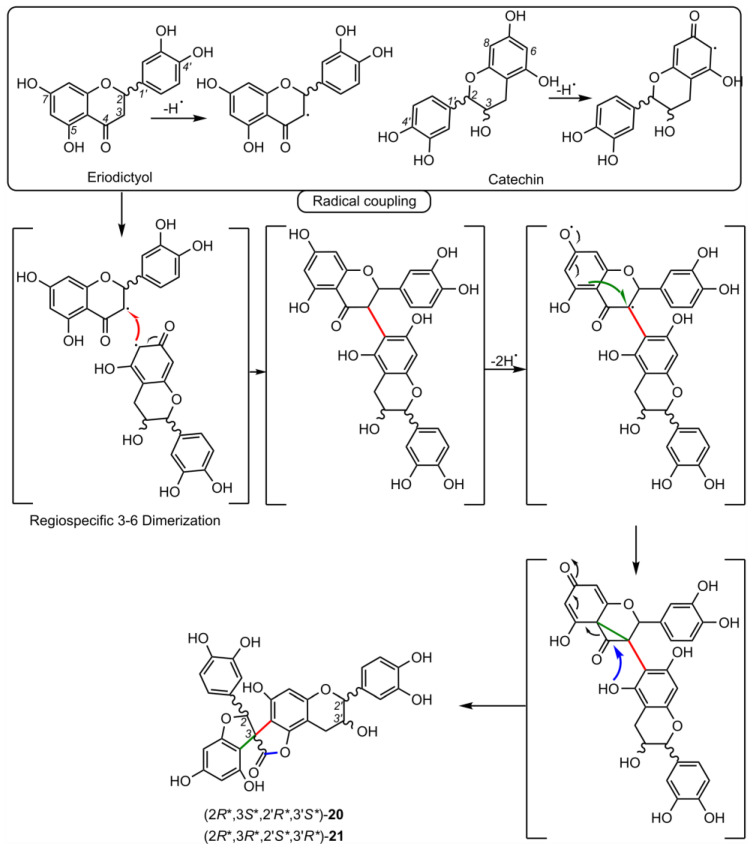
Hypothetical biosynthetic routes to olgensisinols C and D.

Olgensisinols C and D (**20**–**21**) can be formed similarly, the only difference being that the initial radical coupling requires a 3–6 linkage (Scheme 2).

**Scheme 3:**
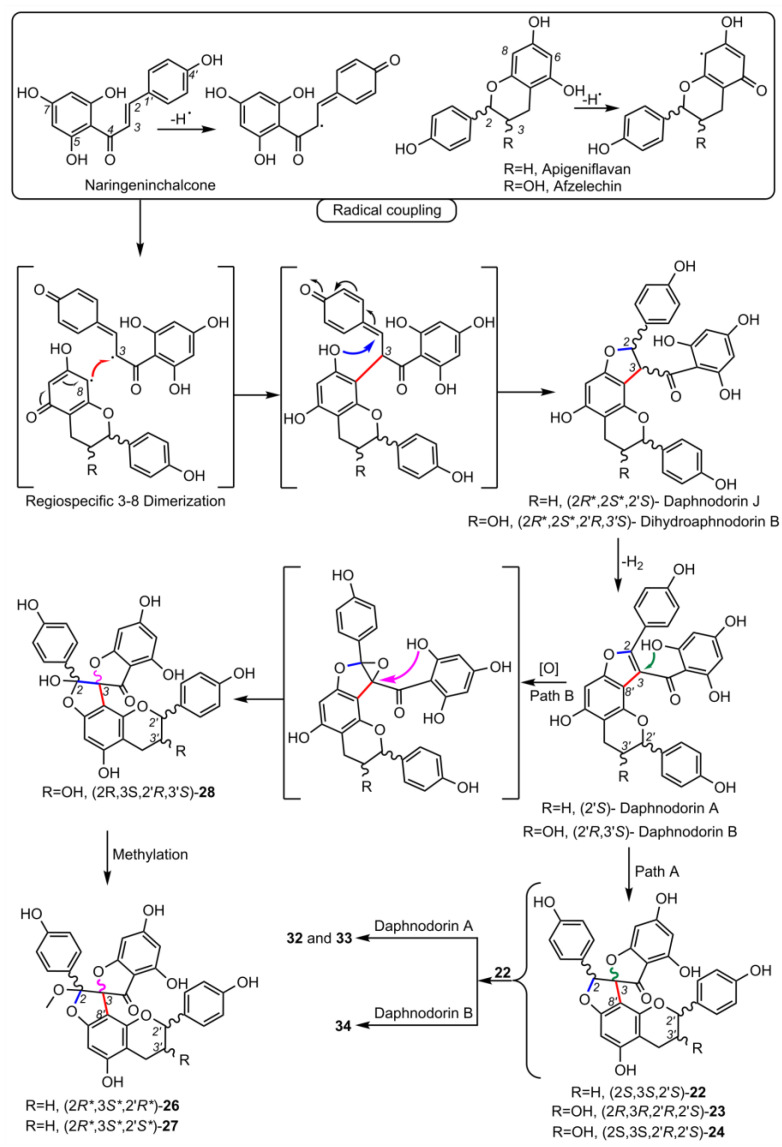
Hypothetical biosynthetic routes to daphnodorin C-type spiro-flavonoids.

The biosynthesis of the spiro-biflavonoids of the daphnodorin C type (Scheme 3), on the other hand, appears to proceed in a slightly different manner and is initiated by the coupling of the naringeninchalcone radical with the apigeniflavan or afzelechin radical (flavan-3-ol). This results in the formation of an activated *p*-methide quinone, which then undergoes nucleophilic substitution with a hydroxyl group to give a benzofuran ring and two known compounds, daphnodorins B and J. These in turn can be dehydrogenated to the next known compounds, daphnodorins A and B, which undergo two divergent reactions (paths A and B). Path A involves cyclization through the attack of the hydroxy group on the C-3 of the double bond, producing compounds **22**–**24**, while path B involves epoxidation of the double bond C-2/C-3 and subsequent opening of the oxirane ring and simultaneous cyclization to a spiro-type system (**28**).

**Scheme 4:**
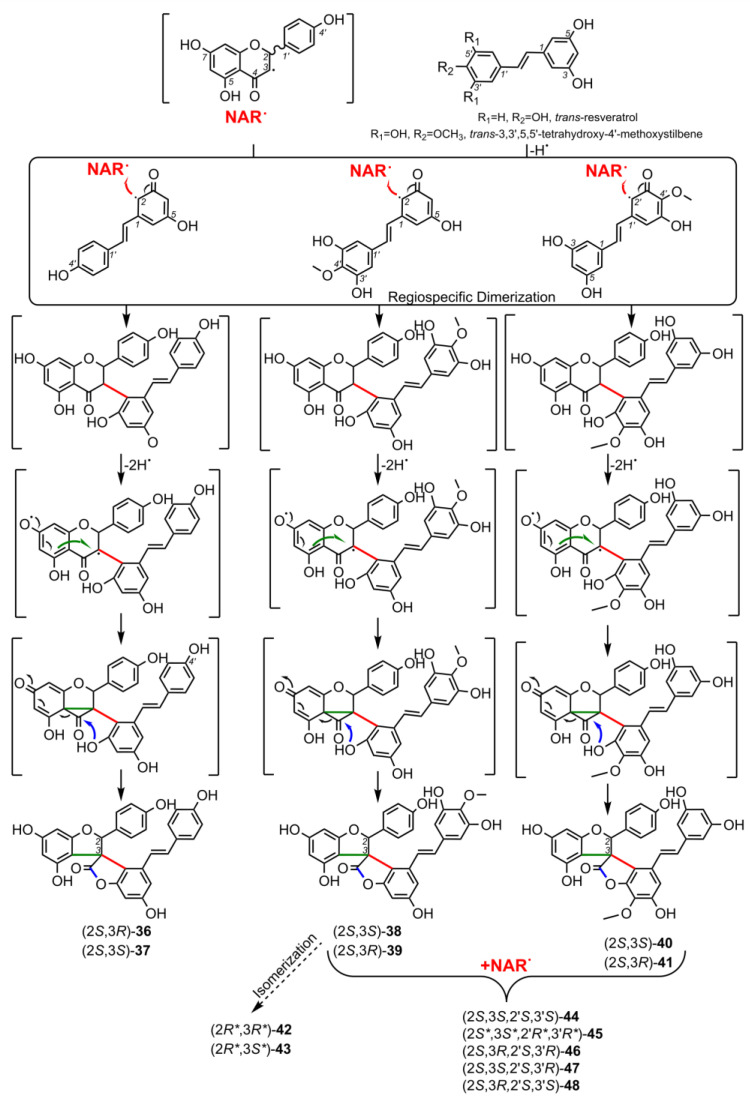
Hypothetical biosynthetic routes to spiro-flavostilbenoids (adapted from Pecio et al. (2023) [7]).

It also appears that the mechanism behind the biosynthesis of the spiro-flavostilbenoids is similar to the one shown in Scheme 1 and Scheme 2. That is, diastereoisomeric pairs of compounds **36**–**37**, **38**–**39**, and **40**–**41** are formed as a result of a regiospecific coupling of the naringenin radical with a stilbene-type radical (resveratrol or THMS). Interestingly, compounds **38**–**41** undergo further coupling with the naringenin radical to give compounds **44**–**48** and probably also form more complex polymeric structures [106] (Scheme 4).

**Scheme 5:**
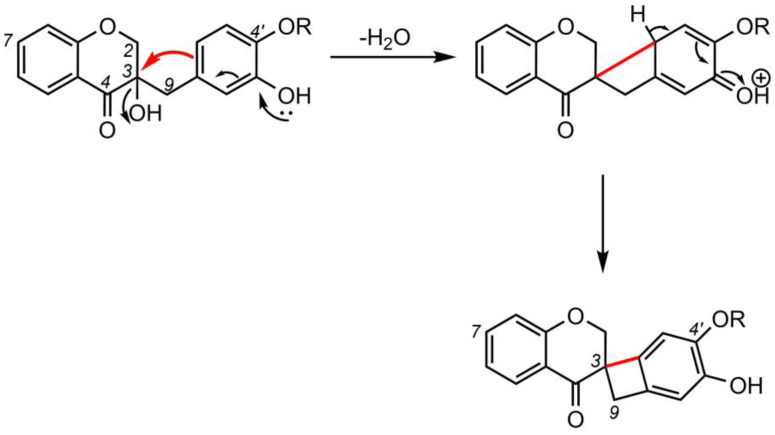
Hypothetical biosynthetic routes to scillascillin-type homoisoflavonoids (adapted from P.M. Dewick (1975) [109]).

The hypothetical mechanisms of homoisoflavonoids formation are described in detail in the work of Castelli & Lopez [107], to which the reader is referred. However, the only works devoted to the study of the biosynthesis of these compounds (specifically, 3-benzylchroman-4-one eucomin from *Eucomis bicolor*) was presented by Dewick [108,109], who showed, by means of feeding experiments with isotopically labeled DL-phenylalanine-[1-^14^C, L-phenylalanine-[U-^14^C], sodium acetate-[2-^14^C], and L-methionine-[*methyl*-^14^C], that 2′-methoxychalcones are biosynthetic intermediate precursors of the homoisoflavonoids sappanin-, scillascillin-, and brazilin-type. The results showed that the C_6_-C_3_ unit from phenylalanine was completely incorporated into the eucomin molecule, becoming the C-4, C-3, C-9, and the aromatic B ring, while the *O*-methyl on the B ring originated from methionine, which was also the source of C-2 (in the pyran ring C). The A-ring, on the other hand, was entirely from acetate. There is one difference from the biosynthesis of flavonoids—an additional atom derived from methionine, which is localized as a methyl group in the 2′-methoxychalcone molecule. The proposed mechanism for the formation of homoisoflavonoids from 2′-methoxychalcone included several oxidation steps necessary to activate the appropriate positions in the molecule for the chemical sense of a given transformation. However, what is missing from the literature is any indication of what processes, either enzymatic or abiotic, are responsible for the transformation of these chalcones into the corresponding homoisoflavonoids. Nevertheless, Dewick suggested that the formation of the 3-*spiro*-cyclobutene ring in scillascillin-type homoisoflavonoids may be related to the transformation of the corresponding sappanin-type homoisoflavonoid [109] (Scheme 5).

## 6. Biological Activities of Spiro-Flavonoids

In recent years, interest in spiro-compounds as drug candidates has increased [4,110,111,112]. Spiro-derivatives were shown to exert a broad range of biological activities, such as antioxidant, anti-inflammatory, neuroprotective, antimicrobial, antidiabetic, anticancer/cytotoxic, antiviral, antimalarial, etc. [4,106,110,112,113,114]. Their pharmacological potential is attributed directly to their inherent three-dimensionality, versatility, rigidity of spiro-cyclic scaffolds, good balance between flexibility and conformational restriction, and structural similarity to important pharmacophore centers [110,113,115]. Spiro-scaffolds are often developed successfully as drug candidates compared to compounds with too many flat aromatic rings (heterocycles) [110,113,115]. However, there is a gap in the synthesis of diverse, architecturally complex spiro-flavonoids. Therefore, the biological activities described here refer to compounds of natural origin. Table S1 (Supplementary Materials) details information on the main biological activities, along with the models used and test results for each substance.

### 6.1. Antioxidant Activity

The antioxidant capacity of the spiro-biflavonoid larixinol (**1**) originating from *Larix decidua* bark was evaluated in vitro using the DPPH assay (2,2-diphenyl-1-picrylhydrazyl) [43]. Compared to reference substances, larixinol was three times less active. Piacente et al. (2004) and Bassarello et al. (2007) tested the ability of **1** and yuccaols A-E (**36**–**40**), yuccaone A (**29**) from *Yucca schidigera* bark, and gloriosaols A-E (**44**–**48**) from *Y. gloriosa* root to scavenge the ABTS^•+^ radical (2,2′-azino-bis(3-ethylbenzothiozoline-6-sulfonic acid) diammonium salt) [28,33]. Their activity was expressed as Trolox Equivalent Antioxidant Capacity (TEAC) values and compared to quercetin (positive control) (Table 3). Comp. **44**–**46**, and a mixture of gloriosaol D and E (**47** and **48**), showed the best antioxidant capacity in this study, much higher than quercetin [28]. Moderate ABTS^•+^ scavenging was observed for the dimeric spiro-flavonoids studied, with activity decreasing in the following order: yuccaol E, larixinol, yuccaol C, yuccaol D, and the weakest for yuccaol A and B, and yuccaone A (**40**, **1**, **38**, **39**, **36**, **37**, **29**, respectively) [33]. Compounds **36**–**40** in β-carotene/linoleic acid autoxidation assay showed significant activity, greater than the positive control—2,6-di-*tert*-butyl-4-methoxyphenol at 120 min [33]. Isolates **1**, **29** were not as active as positive control in this assay [33].

The 15-LOX (lipoxygenase) inhibition assay was used to test the ability of **37** and **44** isolated from the bark of *Y. schidigera* to protect polyunsaturated acids from peroxidation [106]. Their antioxidant activity was higher (EC_50_ = 9.66 µg/mL for yuccaol B, EC_50_ = 12.34 µg/mL for gloriosaol A) compared to the positive control: ascorbic acid (EC_50_ = 21.52 µg/mL) [106].

Nishida et al. (2013) [116] investigated antioxidant activities in DPPH (Trolox, curcumin, and α-tocopherol as positive controls), hydrogen peroxide (H_2_O_2_; Trolox as a positive control), and nitric oxide (NO; Trolox and curcumin as positive controls) scavenging assays of scillascillin-type homoisoflavonoids **49**, **51**, **52**, and **59** isolated from bulbs of *Scilla scilloides*. Isomuscomosin (**52**) tested at 500 μM showed more than 90% DPPH radical scavenging activity (EC_50_ = 22.9 μM; the other compounds tested were below 40%). In the H_2_O_2_ assay, scillascillin (**49**) and 2-hydroxy-scillascillin (**51**) showed activity below 40%, while isomuscomosin (**52**) and scillavone A (**59**) were highly active at 99.3% and 85.4%, respectively. In NO assays, the compounds tested were inactive (below 40% inhibition).

### 6.2. Anti-Inflammatory Activity

Li et al. (2009) investigated the ability of spiro-biflavonoids (**1–3**) to reduce the level of NO production in macrophage cell line RAW 264.7, induced by lipopolysaccharide (LPS) [39]. Only larixinol showed NO inhibitory activity at 100 µg/mL with and IC_50_ value of 60.0 μg/mL [39]. Daphnodorin C and I (**22**, **24**) and 2″-methoxy-daphnodorin C and 2″-methoxy-2-*epi*-daphnodorin C (**26**, **27**) were tested for inhibitory activity against LPS-induced NO production in RAW 264.7 macrophages [49,50]. Only **26** and **27** showed statistically significant inhibitory activity of 32% and 58%, respectively, against an increase of NO at a concentration of 100 µg/mL (compared to 50% for the positive control, aminoguanidine at 25 µM). Compounds **22** and **24**, isolated from the stems of *Daphne kiusiana* Miq., were investigated for their anti-inflammatory potential in the treatment of chronic obstructive pulmonary disease [52]. Comp. **22** most effectively suppressed the inflammatory response in both in vitro (phorbol 12-myristate 13-acetate)-stimulated human lung epithelial cells NCI-H292 and in an in vivo chronic obstructive pulmonary disease model, using mice exposed to cigarette smoke and LPS. Daphnodorin C (**22**) negatively affected the expression of inflammatory genes by inhibiting nuclear factor kappa light chain enhancer of activated B cells (NF-κB) and specific MAPK signaling pathways (mitogen-activated protein kinases) (JNK and p38) and suppressed reactive oxygen species (ROS) products in vitro and in vivo. Daphnodorin C at 20 mg/kg was comparable to the positive control roflumilast at 5 mg/kg [52].

A series of spiro-flavostilbenoids (yuccaols A-E, **36**–**40**) from *Y. schidigera* bark was tested for anti-inflammatory activity using in vitro assays of COX-1, COX-2, and LTB_4_ (leukotriene B4) formation mediated by 5-LOX. Comp. **36** and **37**, containing resveratrol as a stilbenic moiety, showed the highest inhibition against COX-1 and moderate inhibition against COX-2 [117]. Comp. **40**, which has a THMS moiety in its structure, expressed a slightly lower COX-1 and COX-2 inhibitory activity than comp. **36** and **37**. The investigated spiro-flavostilbenoids did not inhibit the formation of LTB_4_ [117]. In another in vitro study, yuccaols A-C (**36**–**38**) were tested on the J774.A1 murine macrophage cells activated with *Escherichia coli* LPS [118]. The anti-inflammatory effect was observed only for compounds **36** and **38** when they were added 1 h before LPS stimulation. Yuccaol C showed the highest activity. It significantly inhibited the iNOS (inducible nitric oxide synthase) protein and NO formation through NF-κB deactivation in a dose-dependent manner, while yuccaol A inhibited significantly only NO release at the highest concentration [118]. Spiro-flavostilbenoids **38**–**43** were evaluated for anti-inflammatory activity on the mouse macrophage cell line RAW 264.7 [11]. Cells were preincubated with the compounds at 100 µM for 1 h and then stimulated with LPS. Yuccalide B (**42**), yuccaol C (**38**), and yuccaol E (**40**), which had the same relative configuration, effectively suppressed the mRNA level of iNOS. Yuccaols C-E significantly reduced transcription of the inflammatory cytokines IL-6 and IL-1β [11].

Waller et al. (2013) tested a series of homoisoflavonoids (at 10 μM) isolated from *Ledebouria socialis* and *L. ovatifolia*, including socialinone (**63**) and C-2 acetylated forms of 2-hydroxy-7-*O*-methyl-scillascillin (**50**) for the inhibitory activity of COX-1 (SC-560 as a positive control) and COX-2 (DuP-607 as a positive control) [22]. These compounds were found to be inactive in COX-2 assay. Nishida et al. (2014) [119] investigated the anti-inflammatory properties of scillascillin-type homoisoflavonoids from bulbs of *Scilla scilloides*, **49**, **51**, **52**, and **59**, using lipoxygenase (nordihydroguaiaretic acid as a positive control) and hyaluronidase (tannic acid as a positive control) as a model of in vitro inflammation. However, the compounds were not active at any of the concentrations tested (500, 750, and 1000 μM). Furthermore, the authors tested the anti-inflammatory effect of scillascillin-type homoisoflavonoids on LPS-activated RAW 264.7 mouse macrophages. Compound **59** showed weak inhibitory activity at 10 μM, while **49**, **51**, **52**, and **59** inhibited NO production level up to 50% at 50 μM. In another work [120], isomuscomosin (**52**) and compound **56** showed similar moderate anti-inflammatory activity in a microsomal fraction assay, while scillascillin (**49**) showed low activity (at concentrations of 250 μg/mL against indomethacin as a positive control). These compounds were found to be inactive in the COX-1 and COX-2 assays. Protosappanin D (**65**) and other constituents of *Caesalpinia sappan* were investigated using an in vitro assay with the J774.1 cell line [36]. Their inhibitory effects on NO and prostaglandin E_2_ (PGE_2_), as well as their suppressive effects on the expression of tumor necrosis factor-α (TNF-α), IL-6, COX-2, and iNOS mRNA level, were evaluated. As a result, **65** inhibited both NO (IC_50_ = 9.6 μM) and PGE_2_ (IC_50_ = 7.8 μM) production. It showed the strongest suppression of TNF-α (IC_50_ = 14.2 μM), IL-6 (IC_50_ = 3.0 μM), COX-2 (IC_50_ = 21.4 μM), and iNOS mRNA expression (IC_50_ = 13.2 μM) among the substances tested.

### 6.3. Neuroprotective Activity

Spiro-flavostilbenoids (**36**–**41**, **44**, **46**–**48**) and spiro-biflavonoids (**6**, **7**) from the bark of *Y. schidigera* were tested in vitro for their inhibitory activity against cholinesterases [7,31]. Acetylcholinesterase (AChE) from electric eel and butyrylcholinesterase (BChE) from horse serum were used in a modified Ellman spectrophotometric assay [7,31]. Compared to galantamine (positive control), the compounds tested showed moderate or weak inhibition of AChE. However, yuccaol B (**37**) and gloriosaol A (**44**) were the most potent inhibitors of BChE, with IC_50_ lower than the positive control (81.3 μM for **37**, 64.9 μM for **44**, and 124.0 μM for galantamine) [7]. Among yuccaols, the (2*S*,3*S*) diastereoisomers showed the highest anticholinesterase activity. The molecular interactions between the most active compounds (**37**, **44**) and human AChE/BChE were studied in silico. These compounds interacted similarly with the peripheral anionic site of AChE and were located deep in the catalytic site of BChE [7]. The neuroprotective effect of yuccaol B and gloriosaol A was also confirmed in vivo using adult zebrafish models [106]. The Y-maze test was used to assess the function of spatial working memory function, while the novel tank diving test was used to measure anxiety in *Danio rerio*. Spiro-flavostilbenoids **37** and **44** at doses of 1, 3, and 5 µg/L attenuated scopolamine-induced amnesia and anxiety, and improved precognitive and anxiolytic activities, to the level of the control group (untreated with scopolamine) [106].

### 6.4. Anticancer and Antitumor Activity

Spiro-biflavonoids abiesinols A-F (**1**, **4**, **12**–**15**), derived from the bark of *Abies sachalinensis*, were screened in vitro for antitumor-initiating activity, and then one of the most active compounds (**12**) was tested in vivo for a skin cancer chemopreventive effect [121]. Their inhibitory effect on the activation of NOR 1 ((±)-(*E*)-methyl-2-[(*E*)-hydroxyimino]-5-nitro-6-methoxy-hex-3-enamide), a donor of nitric oxide, was evaluated in *Chang* human liver cells, using curcumin as a reference compound. The inhibitory activity of all compounds tested was similar to that of curcumin; only abiesinols E (**1**) and F (**4**), which have fewer OH groups in their structure than abiesinols A-D, were slightly less active. A two-step skin carcinogenesis assay was performed using specific-pathogen-free female ICR mice (6 weeks old). Abiesinol A (**12**) was administered orally (0.0025% of body weight) for 2 weeks; skin carcinogenesis was induced with a single dose of peroxynitrite (ONOO^-^) and promoted with 12-*O*-tetradecanoylphorbol-13-acetate applied topically twice a week for 20 weeks [121]. Compound **12** showed significant antitumor-initiating activity in the in vivo test, the percentage of papilloma bearing mice was reduced to 33% at week 11, tumor formation was delayed by 2 weeks, and at the end of the experiment the average number of papillomas per mouse was reduced by a factor of 2, compared to the control group (no abiesinol A treatment) [121]. In contrast, the spiro-biflavonoids 3-*epi*-larixinol (**2**) and fragranols B and C (**8**, **9**), as well as the spiro-triflavonoid fragranol A (**30**), were not active against the human pancreatic cancer cell line PANC-1 in an in vitro assay. Compounds **2**, **8**, **9**, and **30** did not kill tumor cells at the maximum tested amount of 100 μM [37,38]. Spiro-biflavonoid genkwanol A (**23**), isolated from the aerial part of *Fumana procumbens* (Dunal) Gren. & Godr., was evaluated in vitro for its anticancer activity against A549 (adenocarcinomic human basal alveolar epithelial cell line), MCF-7 (human breast cancer cell line), HeLa (human cervical cancer cell line), and BEAS-2B (human bronchial epithelial cell line) cells using the MTT (3-(4,5-dimethylthiazol-2-yl)-2,5-diphenyl-2*H*-tetrazolium bromide) assay [34]. Compound **23**, at the highest concentration tested (20 μg/mL), was active only against A549 cells, showing a 16.8% inhibition of cell viability [34]. Similarly, **23** had a moderate antimitotic effect, compared to the positive controls of colchicine and vinblastine in the microtubule polymerization bioassay in vitro [61]. In another work, the affinity of **23**, daphnodorin I (**24**), 2″-hydroxygenkwanol A (**28**), and 4′-methylgenkwanol A (**25**) to Hsp90 protein, one of the most promising targets for anticancer therapy, was tested in vitro [54]. All spiro-biflavonoids interacted with the immobilized protein, but comp. **28** was the most efficient [54].

Spiro-flavostilbenoids **36**–**38** inhibited Kaposi sarcoma (KS) cell proliferation, migration, and synthesis of the inflammatory mediator PAF (platelet-activating factor) in vitro [122]. Yuccaol C (**38**) was the most active, and it completely blocked the growth of the VEGF (vascular endothelial growth factor)-induced cells, more efficiently attenuated p38 and p42/44 MAP kinase signaling pathways activated by VEGF, and completely suppressed cell migration of KS cells after the PAF treatment. Moreover, yuccaols A-C inactivated VEGF-induced PAF biosynthesis through acetyl transferase blockade and enhanced PAF degradation through lysophospholipid transacetylase activation [122]. The cytostatic and pro-apoptotic activities of gloriosaols A-C (**44**–**46**) were tested in vitro against different cancer cell lines: MCF7 (breast carcinoma), HepG2 (hepatoblastoma), U937 (monocytic leukemia), Molt4 (lymphoblastic leukemia), and Jurkat (T-cell leukemia) [123]. Inhibition of cancer cell growth was observed after 24 h treatment with increasing concentrations of **44**–**46**. These stereoisomers showed different antiproliferative potentials: **46** had the lowest EC_50_ values, and the best effect was observed towards the U937 cell line, followed by **44** and **45** [123]. At the highest doses of 10–25 µM, gloriosaol C (**46**) induced apoptosis in the most sensitive cell line, U937, and tended to switch to necrosis at doses above 30 µM. However, the cytotoxic (proapoptotic) effect of **46** against human peripheral blood mononuclear cells was distinctly weaker. Gloriosaol C-induced apoptosis at doses > 10 µM caused mitochondrial depolarization and cytochrome *c* release in U937 cells [123]. The **44**–**46** tests altered the intracellular redox balance by increasing ROS (reactive oxygen species) release in U937 cancer cells exposed to these compounds for 1 h, and reducing ROS levels in cells simultaneously exposed to a pro-oxidant agent; that is, *t*-butyl hydroperoxide. The best pro-oxidant and antioxidant effect was observed for **46** at doses higher than 10 µM. This is one of the mechanisms by which these stereoisomers (**44**–**46**) were able to induce apoptosis in U937 cells. [123].

Schwikkard et al. (2019) [124] reported the antiproliferative activity of several structurally diverse homoisoflavonoids (including **49**, **52**, **53**, **63**, and **64**) from *Ledebouria ovatifolia* and *Chionodoxa luciliae* Boiss. against endothelial tumor cells (human retinal microvascular endothelial cells) and ocular tumor cells (uveal melanoma 92-1 and retinoblastoma Y79). The authors indicate that the activity of homoisoflavonoids tested was generally related to the substitution pattern of their A and B rings. However, the presence of the spiro-ring rendered the tested scillascillin-type homoisoflavonoids completely inactive, regardless of the substituents in the A and B rings. Likewise, Matsuo et al. (2014) [13] evaluated (3*R*)-5,7-dihydroxy-6-methyl-3-(3′-hydroxy-4′-methoxybenzyl)chroman-4-one and compound **57** (isolated from *Bessera elegans*), which differed only in the presence of the 3-*spiro*-cyclobutene ring (formed by the coupling of C-3 and C-2′ carbons), for cytotoxicity against human HL-60 promyelocytic leukemia cells and normal human diploid TIG-3 fibroblasts. The homoisoflavonoid without the cyclobutene ring showed a potent tumor-selective cytotoxic activity against HL-60 cells, while **57** was noncytotoxic (and noncytotoxic against TIG-3). On the other hand, Chinthala et al. (2014) [21] performed an in vitro anticancer assay using scillascillin (**49**) isolated from *Ledebouria hyderabadensis* against the human cancer cell lines MCF-7 (breast cancer) and DU-145 (prostate cancer). Scillascillin showed significant activity (IC_50_ of 9.59 ug/mL and 11.32 μg/mL, respectively). Doxorubicin was used as a positive control. Its IC_50_ against the tested cell lines was 1.86 and 13.71 μg/mL, respectively.

### 6.5. Cytotoxicity/Mutagenicity

The lack of cytotoxicity of spiro-biflavonoid larixinol (**1**) at a dose of 100 µg/mL was confirmed in the RAW264.7 macrophages using the MTT assay [39]. No cytotoxicity of daphnodorin C (**22**) and daphnodorin I (**24**) at concentrations of 2.5–20 µM was detected against NCI-H292 human lung epithelial cells using the Cell Counting Kit-8 assay [52]. Spiro-flavostilbenoids **36–38** (yuccaols A-C) showed no toxicity against J774.A1 murine monocyte/macrophage cells at doses of 0.1–100 µM in an MTT test [118]. These compounds, at doses of 10–500 µg, confirmed their non-toxicity and demonstrated their non-mutagenicity in *Salmonella typhimurium* strains TA97, TA98, TA100, and TA102 tested by the microsome test (Ames test) [125].

### 6.6. Antiplatelet Activity

Sakuma et al. (1998) [126] tested spiro-biflavonoid daphnodorin C (**22**) isolated from *Daphne odora* roots for the activities of 12-lipoxygenase (12-LOX) and cyclooxygenase (COX). 12-LOX converts arachidonic acid to 12-hydroperoxy-5,8,10,14-eicosatetraenoic acid, which is subsequently reduced to 12-hydroxy-5,8,10,14-eicosatetraenoic acid (12-HETE). 12-HETE has been reported to induce platelet aggregation. COX, on the other hand, produces prostaglandin endoperoxides. These are converted by thromboxane synthase to 12-hydroxy-5,8,10-heptadecatrienoic acid (HHT). Daphnodorin C (**22**) caused 34.1% inhibition of 12-HETE formation and 26.6% inhibition of HHT at a concentration of 100 μM. This makes **22** a dual inhibitor of 12-LOX and COX in platelets.

In vitro, the spiro-flavostilbenoids **36** and **38** yuccaols A and C showed an inhibitory effect on platelet aggregation induced by thrombin and ADP (adenosine diphosphate) in a dose-dependent manner. Pig blood platelets pretreated with the compounds **36** and **38** at their highest concentration (25 μg/mL) caused a suppression of thrombin-induced aggregation by about 50% [127].

### 6.7. Antidiabetic Activity

A series of spiro-biflavonoid diastereoisomers (**1**–**4**, **10**, **11**) isolated from the stem bark of *Glyptostrobus pensilis* were screened in vitro for their ability to inhibit human protein tyrosine phosphatase 1B (PTP1B). This enzyme negatively regulates insulin signaling or epidermal growth factor pathways [35]. Spiropensilisol A (**10**) showed the best activity, inhibiting the enzyme to the same extent as a positive control (oleanolic acid) with an IC_50_ =3.3 µM. The remaining compounds tested were also active, with an IC_50_ ≤ 17.1 µM. Using a molecular modeling approach, compound **10** was shown to interact with the catalytic site of the PTP1B enzyme [35]. Daphnodorins C and I (**22**, **24**) and spiro-tetraflavonoids edgechrins A, B, and D (**32**, **34**, **33**) isolated from *Edgeworthia chrysantha* Lindl. were tested for α-glucosidase inhibitory activity, using *p*-nitrophenyl α-D-glucoside as substrate [58]. All compounds significantly inhibited α-glucosidase, but dimeric compounds **32**–**34** had a stronger activity than **22** and **24** (IC_50_ 1.09, 0.96, 2.13 versus 4.00 and 19.0, respectively, with 73.6 μM for the positive control, acarbose).

### 6.8. Antibacterial, Antifungal, and Antiviral Activity

Inamori et al. (1987) [128] tested the antifungal activity of spiro-biflavonoid daphnodorin C (**22**) against pathogens including *Pyricularia oryzae*, *Rhizoctonia solani*, *Phytophora infestans*, *Botrytis cinerea*, *Puccinia recondita*, and *Erysiphe graminis*, and its insecticidal activity (against *Spodoptera litura*, *Nilaparvata lugens*, *Callosobruchus chinensis*, and *Tetranychus urticae*). It showed antifungal activity only against *P. oryzae* associated with *Oriza sativa* as a plant host. The leaves of rice plant were incubated with daphnodorin C for 3 d before being inoculated with fungal spores for 5 d. The protective value of **22** was 89–90% at a concentration of 200–500 ppm. The insecticidal activity was negligible. Genkwanol A (**23**) caused an in vitro morphological deformation of the phytopathogenic fungus responsible for rice blast—*P. oryzae.* Compared to antifungal controls, that is, griseofulvin and nocodazole, **23** had lower minimum morphological deformation concentration [61].

Daphnodorin C (**22**), isolated from the bark of *Dahpne odora* Thunb., showed moderate inhibition of HIV-1 replication in MT-4 cells [129]. Compared to a positive control, 2′,3′-dideoxycytidine- 5′-triphosphate, comp. **22** had a weak inhibitory effect on HIV-1 reverse transcriptase. The authors suggest that daphnodorin C has anti-HIV-1 effects through inhibition of the early stage of viral replication [129]. Genkwanol A (**23**), derived from the root of *Wikstroemia indica* (L.) C.A.Mey., showed moderate activity against human immunodeficiency virus type 1 (HIV-1) in T4 lymphocytes (CEM cell line) [61].

Antibacterial activity of scillascillin-type homoisoflavonoids **49**, **52**, and **56** isolated from *Drimiopsis maculata* Lindl. & Paxton, and *Eucomis schijffii* Reyneke, was screened against *Staphylococcus aureus*, using the bioautographic and microplate assays [130]. Significant inhibitory activity was obtained for scillascillin (**49**), with a minimum inhibitory concentration (MIC) value of 0.50 mM (neomycin, positive control, had MIC = 0.0025 mM), while compound **52** (isomuscomosin) exhibited bacteriostatic activity with a bacteriostatic concentration value of 1.97 mM.

### 6.9. Phytotoxic Activity

Spiro-biflavonoid genkwanol A (**23**), isolated from the roots of *Stellera chamaejasme* L., showed strong phytotoxic activity against *Arabidopsis thaliana* seedlings, although only at relatively high concentrations (200 μg/mL), with an IC_50_ of 74.8 μg/mL, by inhibiting root growth [59]. This was confirmed by measuring the level of endogenous auxin in the root tip of the transgenic *A. thaliana DR5::GUS* line. This level was significantly reduced by **23** as a result of inhibition of auxin transport. At the same time, comp. **23** was not secreted into the soil by the roots of *S. chamaejasme*. Therefore, it is unlikely that it is responsible for the allelopathic effect exerted by this plant in nature.

### 6.10. Other Activities

Angiotensin II plays a key role in regulating blood pressure, and Takai et al. (1999) [131] tested the inhibition of human chymase-dependent angiotensin II-forming activity by daphnodorin A, daphnodorin B, and daphnodorin C (**22**) isolated from *D. odora*. The compound did not inhibit chymase-generated angiotensin II formation, but also did not affect the formation of angiotensin-converting enzyme-dependent angiotensin II and, unlike daphnodorin A, did not inhibit purified human tryptase.

The spiro-biflavonoids genkwanol A (**23**) and 2″-hydroxygenkwanol A (**28**) were tested in vitro for poly(ADP-ribose) polymerase 1 (PARP-1) inhibitory activity [132]. PARP-1 is an enzyme involved in the pathogenesis of cancer, inflammation, diabetes, and neurodegenerative diseases. Compound **28** binds efficiently to the PARP-1 protein catalytic domain in the nicotine binding pocket. 2″-Hydroxygenkwanol A strongly inhibited PARP-1 activity at submicromolar concentrations at a level comparable to the positive control—3-aminobenzamide [132].

Fusi et al. (2010) [17] investigated the vasorelaxant effects of scillascillin-type homoisoflavonoids **49**, **52**, and **56** obtained from *D. maculata* and *E. schijffii* using rat aortic ring preparations. The authors reported that both 60 mM K^+^ (K60) and phenylephrine-induced tonic contractions were inhibited, in a concentration-dependent manner, by all homoisoflavonoids tested. Compound **56** was found to be the most effective vasorelaxing agent. This was in part due to the activation of soluble guanylyl cyclase. Sasaki et al. (2010) [133] investigated the vasorelaxant activity of protosappanin D (**65**) isolated from *C. sappan* on the rat aorta and mesenteric artery. The authors reported that **65** exhibited vasorelaxing activity on both phenylephrine-preconstricted blood vessels and that its activity was independent of the aortic endothelium and dependent on the mesenteric artery endothelium. The involvement of NO and prostaglandin as endothelium-derived relaxing factors was demonstrated in further experiments with *N*^G^-nitro-L-arginine and indomethacin.

## 7. Conclusions and Further Directions

Spiro-flavonoids, due to the presence of an unusual structural element such as spiro-carbon, are attracting increasing interest because of their chemical and biological properties. A total of 65 spiro-flavonoid structures which were monomeric, as well as bi-, tri-, and tetrameric, belonging to several groups differing in the type of polyphenolic units and the way they are combined, were isolated and characterized. Spiro-biflavonoids were the most abundant group, most frequently isolated from the families Pinaceae, Thymelaeaceae, Cupressaceae, and Pentaphylacaceae. In turn, the richest source of spiro compounds (thirty-four structures) was the Asparagaceae family, from which all known scillascillin-type homoisoflavonoids and all spiro-flavostilbenoids were derived. Most of the oligomeric spiro-flavonoids were isolated from woody plant parts (twigs, bark, and roots), while monomeric scillascillin-type homoisoflavonoids were obtained from bulbs. Methods used to isolate them mainly included classical extraction by maceration at room temperature using pure organic solvents of relatively different polarity and their mixtures, most often with water. The subsequent separation steps also included classical separation techniques based on the difference in solubility in two immiscible liquids (liquid–liquid extraction), as well as the use of column liquid chromatography in normal and reversed-phase systems and gel filtration. However, normal-phase column chromatography was the most widely used technique. As modern chemistry strives to be as “green” as possible, future studies should use extraction methods based on less toxic solvents, such as CO_2_ supercritical fluid extraction or deep eutectic solvents.

The relative and absolute configurations of the complex structures of spiro-flavonoids, frequently containing multiple chiral carbons (including spiro-carbons), have been determined by a number of spectroscopic techniques, including nuclear magnetic resonance (NMR), electronic circular dichroism (ECD), X-ray diffraction (XRD), and chemical methods using chiral derivatizing agents. NMR and XRD are the methods most commonly used to determine their relative configurations, although an increasing number of cases of the use of quantum mechanical (QM) calculations (e.g., modified DP4+ probability method) are reported in the literature. Empirical methods have been used to assign absolute configuration, including the comparison of Cotton effects between known and newly described compounds. However, this method seems to be far from sufficient for structures with more than one chirality center, so it is necessary to systematically and correctly use QM techniques to predict ECD spectra using time-dependent density-functional theory calculations.

This review also summarizes the topic of possible pathways for spiro-flavonoid biosynthesis. The available knowledge provides some clues pointing towards mechanisms involving radical coupling reactions. Nevertheless, it is noteworthy that there are essentially no works that explore this topic in depth, for example, using isotopic labeling, which we believe should provide a direction for future research.

The potential health benefits of spiro-flavonoids have been summarized and the available results indicate significant anti-inflammatory, neuroprotective, antitumor/anticancer, and antidiabetic properties in vitro and in vivo of some spiro-biflavonoids and spiro-flavostilbenoids. On the other hand, scillascillin-type homoisoflavonoids showed good vasorelaxant activity. Therefore, future research should focus on these aspects of their activity.

## Figures and Tables

**Table 2:** Chemical structures of spiro-biflavonoids.

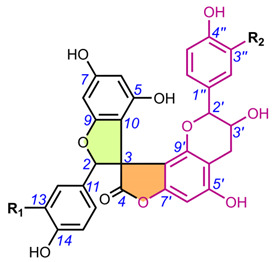					
Compound	Name	R_1_	R_2_		Stereochemistry
C_30_H_22_O_10_ (MW = 542.49)					
**1**	Larixinol (=Abiesinol E)	H	H		(2*R*,3*R*,2′*R*,3′*R*)
**2 ^a^**	3-*Epi*-larixinol	H	H		(2R*,3R*,2′R*,3′S*)
**3 ^a^**	3,2′-*Epi*-larixinol	H	H		(2S*,3R*,2′R*,3′S*)
**4**	Abiesinol F	H	H		(2*R*,3*S*,2′*R*,3′*R*)
**5**	Yuccalechin A	H	H		(2*S*,3*R*,2′*R*,3′*R*)
**6**	Yuccalechin B	H	H		(2*S*,3*R*,2′*R*,3′*S*)
**7**	Yuccalechin C	H	H		(2*S*,3*S*,2′*R*,3′*S*)
**8**	Fragranol B	H	H		(2*S*,3*R*,2′*S*,3′*R*)
**9**	Fragranol C	H	H		(2*R*,3*S*,2′*S*,3′*R*)
**10**	Spiropensilisol A	H	H		(2R*,3S*,2′R*,3′S*)
**11**	Spiropensilisol B	H	H		(2S*,3S*,2′R*,3′S*)
C_30_H_22_O_11_ (MW = 558.49)					
**12 ^a^**	Abiesinol A (=13-Hydroxylarixinol)	H	OH		(2*R*,3*R*,2′*R*,3′*R*)
**13**	Abiesinol B	H	OH		(2*R*,3*S*,2′*R*,3′*R*)
**14**	Abiesinol C (=Olgensisinol A)	OH	H		(2*R*,3*R*,2′*R*,3′*R*)
**15**	Abiesinol D	OH	H		(2*R*,3*S*,2′*R*,3′*R*)
C_30_H_22_O_12_ (MW = 574.49)					
**16**	Abiesinol G (=Vitisinol)	OH	OH		(2*R*,3*R*,2′*R*,3′*R*)
**17**	Abiesinol H	OH	OH		(2*R*,3*S*,2′*R*,3′*R*)
**18**	Larisinol	OH	OH		(2R*,3R*,2′R*,3′S*)
**19**	Olgensisinol B	OH	OH		(2*R*,3*S*,2′*S*,3′*S*)
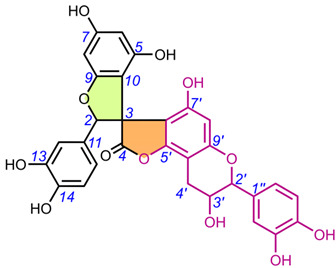					
**Compound**	**Name**	**Stereochemistry**			
C_30_H_22_O_12_ (MW = 574.49)					
**20**	Olgensisinol C	(2R*,3S*,2′R*,3′S*)			
**21**	Olgensisinol D	(2R*,3R*,2′S*,3′R*)			
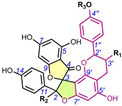					
**Compound**	**Name**	**R_1_**	**R_2_**	**R_3_**	**Stereochemistry**
C_30_H_22_O_9_ (MW = 526.49)					
**22**	Daphnodorin C	H	H	H	(2*S*,3*S*,2′*S*)
C_30_H_22_O_10_ (MW = 542.49)					
**23**	Genkwanol A	OH	H	H	(2*R*,3*R*,2′*R*,3′*S*)
**24**	Daphnodorin I	OH	H	H	(2*S*,3*S*,2′*R*,3′*S*)
C_31_H_24_O_10_ (MW = 556.52)					
**25 ^a^**	4′-Methylgenkwanol A	OH	H	CH_3_	(2*R*,3*S*,2′*R*,3′*S*)
**26 ^a^**	2″-Methoxy-daphnodorin C	H	OCH_3_	H	(2R*,3S*,2′R*)
**27 ^a^**	2″-Methoxy-2-*epi*-daphnodorin C	H	OCH_3_	H	(2R*,3S*,2′S*)
C_30_H_22_O_11_ (MW = 558.49)					
**28 ^a^**	2″-Hydroxygenkwanol A				(2*R*,3*S*,2′*R*,3′*S*)
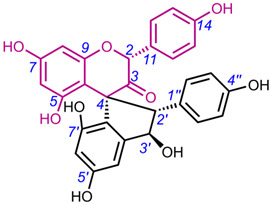					
**Compound**	**Name**	**Stereochemistry**			
C_29_H_22_O_9_ (MW = 514.48)					
**29**	Yuccaone A	(2R*,4R*,2′S*,3′R*)			

^a^ The name of the compound refers to the name given in the original publication, while the names according to the numbering in the figure are: 3′-*epi*-larixinol (**2**), 3′,2-*epi*-larixinol (**3**), 3″-hydroxylarixinol (**12**), 4″-methylgenkwanol A (**25**), 2-methoxy-dpahnodorin C (**26**), 2-methoxy-2′-*epi*-daphnodorin C (**27**), and 2-hydroxygenkwanol (**28**).

**Table 3:** Chemical structures of spiro-triflavonoids.

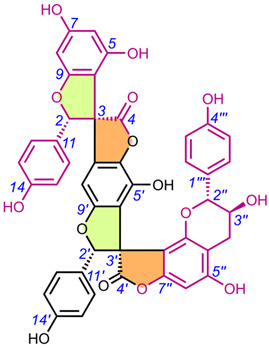		
Compound	Name	Stereochemistry
C_45_H_30_O_15_ (MW = 810.71)		
**30**	Fragranol A	(2*S*,3*S*,2′*S*,3′*R*,2″*R*,3″*S*)
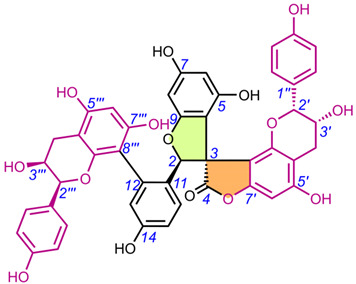		
**31**	Triflarixinol	(2R*,3R*,2′R*,3′R*,2‴S*,3‴S*)

**Table 4:** Chemical structures of spiro-tetraflavonoids.

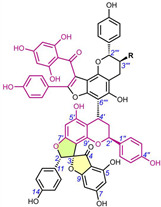			
Compound	Name	R	Stereochemistry
C_60_H_42_O_18_ (MW = 1050.97)			
**32**	Edgechrin A	H	(2S*,3S*,2′S*,4′R*,2‴S*)
C_60_H_42_O_19_ (MW = 1066.97)			
**34**	Edgechrin B	OH	(2S*,3S*,2′S*,4′R*,2‴R*,3‴S*)
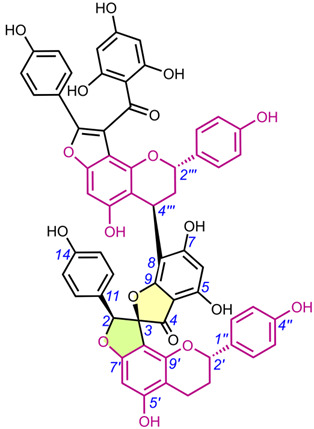			
**Compound**	**Name**		**Stereochemistry**
C_60_H_42_O_18_ (MW = 1050.97)			
**33**	Edgechrin D		(2S*,3S*,2′S*,2‴S*,4‴R*)
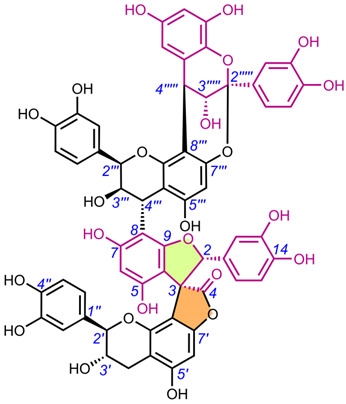			
**Compound**	**Name**		**Stereochemistry**
C_60_H_44_O_24_ (MW = 1148.98)			
**35**	Pinuspirotetrin		(2*R*,3*S*,2′*R*,3′*S*,2‴*R*,4‴*R*,2‴″*S*,3‴″*R*,4‴″*R*)

**Table 5:** Chemical structures of spiro-flavostilbenoids.

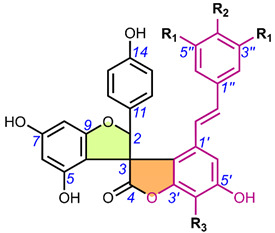					
Compound	Name	R_1_	R_2_	R_3_	Stereochemistry
C_29_H_20_O_8_ (MW = 496.47)					
**36**	Yuccaol A	H	OH	H	(2*S*,3*R*)
**37**	Yuccaol B	H	OH	H	(2*S*,3*S*)
C_30_H_22_O_10_ (MW = 542.49)					
**38**	Yuccaol C	OH	OCH_3_	H	(2*S*,3*S*)
**39**	Yuccaol D	OH	OCH_3_	H	(2*S*,3*R*)
**40**	Yuccaol E	OH	H	OCH_3_	(2*S*,3*S*)
**41**	Yuccalide A	OH	H	OCH_3_	(2*S*,3*R*)
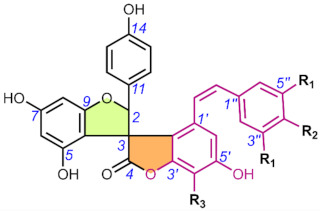					
**Compound**	**Name**	**R_1_**	**R_2_**	**R_3_**	**Stereochemistry**
C_30_H_22_O_10_ (MW = 542.49)					
**42**	Yuccalide B	OH	OCH_3_	H	(2R*,3R*)
**43**	Yuccalide C	OH	OCH_3_	H	(2R*,3S*)
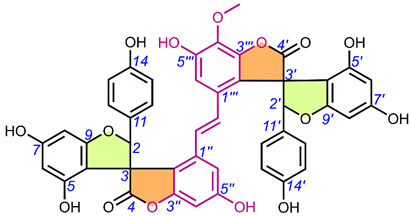					
**Compound**	**Name**	**Stereochemistry**			
C_45_H_30_O_15_ (MW = 810.71)					
**44**	Gloriosaol A	(2*S*,3*S*,2′*S*,3′*S*)			
**45**	Gloriosaol B	(2S*,3S*,2′R*,3′R*)			
**46**	Gloriosaol C	(2*S*,3*R*,2′*S*,3′*R*)			
**47**	Gloriosaol D	(2*S*,3*S*,2′*S*,3′*R*)			
**48**	Gloriosaol E	(2*S*,3*R*,2′*S*,3′*S*)			

**Table 6:** Chemical structures of scillascillin-type homoisoflavonoids. Protosappanin D (**65**) is a dimeric protosappanin-type homoisoflavonoid.

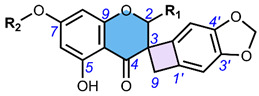								
Compound	Name	R_1_	R_2_	Stereochemistry				
C_17_H_12_O_6_ (MW = 312.27)								
**49**	Scillascillin	H	H	(3*R*)				
C_18_H_14_O_7_ (MW = 342.30)								
**50**	2-Hydroxy-7-*O*-methyl-scillascillin	OH	CH_3_	*rac*-(2R*,3*R*)				
C_17_H_12_O_7_ (MW = 328.27)								
**51**	2-Hydroxy-scillascillin	OH	H	(3R*)				
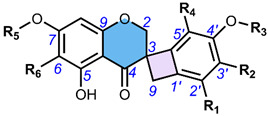								
**Compound**	**Name**	**R_1_**	**R_2_**	**R_3_**	**R_4_**	**R_5_**	**R_6_**	**Stereochemistry**
C_17_H_14_O_6_ (MW = 314.29)								
**52**	3′,5,7-Trihydroxy-4′-methoxyspiro[2*H*-1-benzopyran-3(4*H*),7′-bicyclo[4.2.0]octa[1,3,5]trien]-4-one (=isomuscomosin)	H	OH	CH_3_	H	H	H	(3*R*)
**53**	4′,5,7-Trihydroxy-3′-methoxyspiro[2*H*-1-benzopyran-3(4*H*),7′-bicyclo[4.2.0]octa[1,3,5]trien]-4-one (=muscomosin)	H	OCH_3_	H	H	H	H	(3*R*)
**54**	3′,4′,5-Trihydroxy-7-methoxyspiro[2*H*-1-benzopyran-3(4*H*),7′-bicyclo[4.2.0]octa[1,3,5]trien]-4-one	H	OH	H	H	CH_3_	H	(3*R*)
C_18_H_16_O_6_ (MW = 328.32)								
**55**	5,5′-Dihydroxy-4′,7-dimethoxyspiro[2*H*-1-benzopyran-3(4*H*),7′-bicyclo[4.2.0]octa[1,3,5]trien]-4-one	H	H	CH_3_	OH	CH_3_	H	(3*R*)
**56**	3′,5-Dihydroxy-4′,7-dimethoxyspiro[2*H*-1-benzopyran-3(4*H*),7′-bicyclo[4.2.0]octa[1,3,5]trien]-4-one	H	OH	CH_3_	H	CH_3_	H	(3*R*)
**57**	5,5′,7-Trihydroxy-4′-methoxy-6-methylspiro[2*H*-1-benzopyran-3(4*H*),7′-bicyclo[4.2.0]octa[1,3,5]-trien]-4-one	H	H	CH_3_	OH	H	CH_3_	(3*R*)
**58**	5,7-Dihydroxy-3′,4′-dimethoxyspiro[2*H*-1-benzopyran-3(4*H*),7′-bicyclo[4.2.0]octa[1,3,5]trien]-4-one	H	OCH_3_	CH_3_	H	H	H	(3R*)
C_18_H_16_O_7_ (MW = 344.32)								
**59**	2′,5,7-Trihydroxy-3′,4′-dimethoxyspiro[2*H*-1-benzopyran-3(4*H*),7′-bicyclo[4.2.0]octa[1,3,5]trien]-4-one (=scillavone A)	OH	OCH_3_	CH_3_	H	H	H	(3*R*)
**60**	2′,4′,5-Trihydroxy-3′,7-dimethoxyspiro[2*H*-1-benzopyran-3(4*H*),7′-bicyclo[4.2.0]octa[1,3,5]trien]-4-one	OH	OCH_3_	H	H	CH_3_	H	(3R*)
C_19_H_18_O_6_ (MW = 342.34)								
**61**	5-Hydroxy-3′,4′,7-trimethoxyspiro[2*H*-1-benzopyran-3(4*H*),7′-bicyclo[4.2.0]octa[1,3,5]trien]-4-one	H	OCH_3_	CH_3_	H	CH_3_	H	(3R*)
C_19_H_18_O_7_ (MW = 358.34)								
**62**	5,7-Dihydroxy-2′,3′,4′-trimethoxyspiro[2*H*-1-benzopyran-3(4*H*),7′-bicyclo[4.2.0]octa[1,3,5]trien]-4-one	OCH_3_	OCH_3_	CH_3_	H	H	H	(3R*)
**63**	2′,5-Dihydroxy-3′,4′,7-trimethoxyspiro[4*H*-1-benzopyran-3(2*H*),7′-bicyclo[4.2.0]octa[1,3,5]trien]-4-one (=socialinone)	OH	OCH_3_	CH_3_	H	CH_3_	H	(3R*)
C_20_H_20_O_7_ (MW = 372.37)								
**64**	5-Hydroxy-2′,3′,4′,7-tetramethoxyspiro[4*H*-1-benzopyran-3(2*H*),7′-bicyclo[4.2.0]octa[1,3,5]trien]-4-one	OCH_3_	OCH_3_	CH_3_	H	CH_3_	H	(3R*)
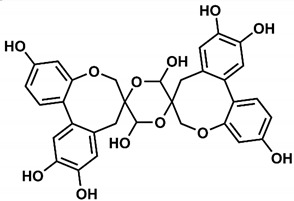								
**Compound**	**Name**	**Stereochemistry**						
C_32_H_28_O_12_ (MW = 604.56)								
**65**	Protosappanin D	Unknown						

**Table 7:** Summary of spiro-flavonoid isolation methods.

Group	Compound	Extraction Solvent	Isolation 1st Step	Isolation 2nd Step	Isolation Final Steps	References
Spiro-biflavonoids	**1**	Acetone (maceration)	LLE H_2_O-CHCl_3_-EtOAc *	CC GPC LH-20 (EtOH)	CC NP Silica gel (CHCl_3_-MeOH)	[45]
	**22**, **24**	Acetone-H_2_O (7:3, *v*/*v*) (maceration, r.t.)	CC RP HP20 (gr. MeOH-H_2_O)	CC GPC LH-20 (gr. MeOH-H_2_O) for **22** or CC RP MCI (gr. MeOH-H_2_O) for **24**	CC RP MCI (MeOH-H_2_O) → CC RP C_8_ (MeOH-H_2_O) for **22** or CC RP HW-40F (MeOH-H_2_O) for **24**	[58]
	**1**, **4**, **12–17**	CHCl_3_ → MeOH (maceration, r.t., successively)	CC RP Diaion HP-20 (MeOH-H_2_O)	CC NP Silica gel (gr. CHCl_3_-MeOH)	MPLC NP Silica gel → CC GPC LH-20 (MeOH) → HPLC RP C_18_ (MeOH-H_2_O)	[42]
	**23**	CHCl_3_-MeOH (2:1, *v*/*v*) (reflux)	CC RP C_18_ (gr. MeOH-H_2_O)	FLASH CC NP Silica gel (gr. CHCl_3_-MeOH-Acetone)	FLASH CC NP Silica gel (Hexanes-EtOAc-MeOH) → CC NP Silica gel (EtOAc-CHCl_3_-MeOH-H_2_O) → HPLC RP C_18_ (CH_3_CN-H_2_O)	[55]
	**1**, **14**, **18**, **22**, **24**	EtOAc (reflux)	CC NP Silica gel (gr. CHCl_3_-MeOH) for **1**, **14**, **18** or CC NP Silica gel (gr. *n*-Hexane-EtOAc) for **22**, **24**	CC NP Silica gel (CHCl_3_-MeOH) or CC GPC LH-20 (MeOH) for **22**	CC GPC LH-20 (MeOH) for **24**	[6,44,56,57]
	**23**	EtOH (r.t.)	LLE H_2_O-Petroleum ether-CHCl_3_-EtOAc	CC NP Silica gel (gr. CHCl_3_-MeOH)		[59]
	**1–3**	EtOH-H_2_O (40%, *v*/*v*) (stirring, r.t.)	HPLC RP C_18_ (CH_3_CN-H_2_O w/FA)			[43]
	**23**	EtOH-H_2_O (7:3, *v*/*v*) (maceration, r.t.)	LLE H_2_O-Petroleum ether-DCM-EtOAc-*n*-BuOH	CC NP Silica gel (gr. Petroleum Ether-EtOAc-MeOH)	CC NP Silica gel (gr. DCM-MeOH) → CC GPC LH-20 (MeOH) → TLC RP (MeOH-H_2_O)	[60]
	**23**	EtOH-H_2_O (75%, *v*/*v*) (reflux)	LLE H_2_O-EtOAc-*n*-BuOH	CC NP Silica gel (gr. CHCl_3_-MeOH)	HPLC RP C_18_ (MeOH-H_2_O)	[61]
	**1–3**	EtOH-H_2_O (80%, *v*/*v*)	LLE H_2_O-CHCl_3_-EtOAc-*n*-BuOH	CC NP Silica gel (gr. CHCl_3_-MeOH)	CC RP C_18_ (MeOH-H_2_O) → CC GPC LH-20 (MeOH)	[39]
	**14**, **16**, **19–21**	EtOH-H_2_O (80%, *v*/*v*)	LLE H_2_O-CHCl_3_-EtOAc	CC RP XAD-7 HP (gr. MeOH-H_2_O)	CC GPC LH-20 (gr. MeOH-H_2_O) → CC RP MCI gel CHP-20P (EtOH-H_2_O) for **14**, **16**, **19** or CC GPC LH-20 (gr. MeOH-H_2_O) → CC GPC LH-20 (MeOH-H_2_O) → CC RP C_18_ (MeOH-H_2_O) for **20**, **21**	[46]
	**16**	EtOH-H_2_O (80%, *v*/*v*)	LLE H_2_O-CHCl_3_-EtOAc-*n*-BuOH	CC RP Diaion HP-20 (MeOH-H_2_O)	CC GPC LH-20 (gr. MeOH-H_2_O) → CC RP MCI CHP-20P (MeOH-H_2_O) → HPLC RP C_18_ (CH_3_CN-H_2_O w/AcOH)	[63]
	**1**, **12**	EtOH-H_2_O (80%, *v*/*v*) (reflux)	LLE H_2_O-CHCl_3_-EtOAc-*n*-BuOH	CC NP Silica gel (gr. CHCl_3_-Acetone)	MPLC RP C_18_ (MeOH-H_2_O) → CC GPC LH-20 (MeOH)	[41]
	**1**	EtOH-H_2_O (85:15, *v*/*v*) (maceration, r.t.)	LLE H_2_O-CHCl_3_-EtOAc-*n*-BuOH	CC NP Silica gel (gr. CHCl_3_-Acetone)	MPLC RP C_18_ (MeOH-H_2_O) → CC GPC LH-20 (MeOH)	[40]
	**2**, **8**, **9**	EtOH-H_2_O (95%, *v*/*v*) (maceration, r.t.)	LLE H_2_O-EtOAc	MPLC NP Silica gel (DCM-EtOAc)	TLC NP Silica gel (DCM-EtOAc)	[38]
	**22**	EtOH-H_2_O (95%, *v*/*v*) (reflux)	LLE H_2_O-Petroleum ether-EtOAc	CC NP Silica gel (gr. DCM-MeOH)	CC NP Silica gel (unknown solvent) → HPLC RP C_18_ (MeOH-H_2_O)	[53]
	**1**, **2**, **22**	EtOH-H_2_O (95%, *v*/*v*) (r.t.)	LLE H_2_O-Petroleum ether-EtOAc-*n*-BuOH	CC NP Silica gel (gr. CHCl_3_-MeOH) for **22** or CC NP Silica gel (Petroleum ether-Acetone) for **1**, **2**	CC GPC LH-20 (CHCl_3_-MeOH) for **22** or HPLC RP C_18_ (MeOH-H_2_O) → CC GPC LH-20 (MeOH) for **1**, **2**	[48,62]
	**22**, **24**, **26**, **27**	MeOH	LLE H_2_O-Petroleum ether-EtOAc-*n*-BuOH	CC NP Silica gel (gr. CHCl_3_-MeOH)	CC RP C_18_ (gr. MeOH-H_2_O) → CC GPC LH-20 (MeOH) or CC GPC LH-20 (MeOH) for **22**, **24**	[49,50]
	**22**, **24**	MeOH(maceration, r.t.)	LLE H_2_O-*n*-Hexane-EtOAc-*n*-BuOH	CC RP C_18_ (MeOH-H_2_O)	HPLC RP C_18_ (CH_3_CN-H_2_O)	[52]
	**29**	MeOH (maceration, r.t.)	CC GPC LH-20 (MeOH)	CC RP C_18_ (CH_3_CN-H_2_O w/H_3_PO_4_)		[32]
	**23**	MeOH (reflux)	CC NP Silica gel (gr. *n*-Hexane-EtOAc → CHCl_3_-MeOH)	CC GPC LH-20 (MeOH-H_2_O)	CC RP MCI CHP 20P (MeOH-H_2_O)	[51]
	**1**	MeOH (r.t.)	CC RP C_18_ (gr. MeOH-H_2_O)	CC RP C_18_ (gr. CH_3_CN-H_2_O)		[33]
	**5–7**	MeOH (r.t.)	LLE H_2_O-*n*-Hexane- EtOAc	CC GPC LH-20 (MeOH)	CC NP Silica gel (gr. CHCl_3_-Acetone-AcOH) → HPLC RP C_18_ (CH_3_CN/MeOH-H_2_O)	[31]
	**23**	MeOH-H_2_O (80%, *v*/*v*) (37 °C)	LLE H_2_O-*n*-BuOH	CC GPC LH-20 (MeOH)	CC NP Silica gel (gr. cyclohexane-EtOAc-MeOH) → CC MPLC RP (CH_3_CN-H_2_O)	[34]
	**1–4**, **10**, **11**	MeOH-H_2_O (90%, *v*/*v*) (r.t.)	CC GPC LH-20	HPLC RP C_18_		[35]
	**23–25**, **28**	*n*-Hexane → CHCl_3_ → CHCl_3_-MeOH (9:1, *v*/*v*) → MeOH (maceration, r.t., successively)	CC GPC LH-20 (MeOH)	HPLC RP C_18_ (MeOH-H_2_O)		[54]
Spiro-triflavonoids	**31**	EtOAc (reflux)	CC NP Silica gel (gr. CHCl_3_-MeOH)	CC NP Silica gel (CHCl_3_-MeOH)		[44]
	**30**	EtOH-H_2_O (95%, *v*/*v*) (maceration, r.t.)	LLE H_2_O-EtOAc	MPLC NP Silica gel (gr. DCM-EtOAc-MeOH)	MPLC NP Silica gel (gr. DCM-MeOH)	[37]
Spiro-tetraflavonoids	**32–34**	Acetone-H_2_O (7:3, *v*/*v*) (maceration, r.t.)	CC RP HP20 (gr. MeOH-H_2_O)	CC GPC LH-20 (gr. MeOH-H_2_O)	CC NP Silica gel (DCM-MeOH) → CC RP HW-40F (MeOH-H_2_O) → CC RP C_8_ (MeOH-H_2_O) → CC RP C_18_ (MeOH-H_2_O) for **32** or CC RP HW-40F (MeOH-H_2_O) → HPLC RP C_18_ (MeOH-H_2_O) → HPLC RP C_18_ (CH_3_CN-H_2_O) for **33** or CC NP Silica gel (DCM-MeOH) → CC RP C_18_ (MeOH-H_2_O) for **34**	[58]
	**35**	Pine bark extract **	LLE H_2_O-EtOAc	CPC Hexane-EtOAc-MeOH-H_2_O (2-4-1-4) → CPC Hexane-EtOAc-MeOH-H_2_O (0.5-4-1-4)	CC GPC LH-20 (EtOH) → CC RP C_18_ (MeOH-H_2_O) → HPLC RP C_18_ (CH_3_CN-H_2_O w/FA)	[47]
Spiro-flavostilbenoids	**38–43**	Acetone (r.t.)	CC RP DMS (gr. MeOH-H_2_O-Acetone)	CC GPC LH-20 (MeOH)	HPLC RP C_18_ (MeOH-H_2_O) → HPLC RP C_18_ (CH_3_CN-H_2_O)	[11]
	**38**, **39**	MeOH	LLE H_2_O-EtOAc-*n*-BuOH	CC NP Silica gel (gr. *n*-Hexane-EtOAc-MeOH)	CC NP Silica gel (*n*-Hexane-EtOAc) → CC GPC LH-20 (MeOH)	[19]
	**36–40**	MeOH (r.t.)	CC RP C_18_ (gr. MeOH-H_2_O)	CC RP C_18_ (gr. CH_3_CN-H_2_O H_3_PO_4_) or CC RP C_18_ (gr. CH_3_CN-H_2_O)		[30,33]
	**41**, **44**, **46–48**	MeOH (r.t.)	LLE H_2_O-*n*-Hexane-EtOAc	CC GPC LH-20 (MeOH)	CC NP Silica gel (gr. CHCl_3_-Acetone-AcOH) → HPLC RP C_18_ (CH_3_CN/MeOH-H_2_O)	[31]
	**38–40**, **44–48**	MeOH-H_2_O (80%, *v*/*v*)	LLE H_2_O-EtOAc	CC GPC LH-20 (MeOH)	HPLC RP C_18_ (gr CH_3_CN-H_2_O w/TFA)	[28,29]
Scillascillin-type homoisoflavonoids	**49**, **52**, **56**	DCM (agitation *or* shaker, r.t.)	CC NP Silica gel (unknown solvent *or* DCM)			[16,23]
	**49**, **50**	DCM → EtOAc → MeOH (shaker, successively)	CC NP Silica gel (*n*-Hexane-DCM-MeOH)	CC NP Silica gel (DCM-Et_2_O)		[22]
	**49**	DCM → MeOH (agitation, r.t.)	CC NP Silica gel (DCM)			[18]
	**63**, **64**	DCM → MeOH (shaker, successively)	CC NP Silica gel (gr. *n*-Hexane-DCM-MeOH)	TLC NP Silica gel (EtOAc-DCM)		[22]
	**50**, **56**, **60–62**, **64**	DCM-MeOH (1:1, *v*/*v*) → MeOH (maceration, r.t., successively)	LLE MeOH-CHCl_3_-H_2_O (lower phase)	CC NP Silica gel (gr. DCM-MeOH)	CC NP Silica gel (gr. CHCl_3_-Light Petroleum) → recrystallization (MeOH) for **50** or CC GPC LH-20 (unknown solvent) → CC NP Silica gel (gr. CHCl_3_-MeOH) → TLC (unknown solvent) for **60** or CC GPC LH-20 (unknown solvent) → CC NP Silica gel (gr. CHCl_3_-Light Petroleum) → TLC (CHCl_3_-Light Petroleum-MeOH) for **56**, **61**, **62**, **64**	[15]
	**53**	DCM-MeOH (1:1, *v*/*v*) → MeOH (maceration, r.t., successively)	LLE (MeOH-H_2_O, 70%, *v*/*v*)-Light Petroleum-CHCl_3_-EtOAc-*n*-BuOH (lower phase)	CC NP Silica gel (gr. Light Petroleum-EtOAc)	CC NP Silica gel (gr. Light Petroleum-CHCl_3_-MeOH)	[15]
	**58**, **61**	DCM-MeOH (1:1, *v*/*v*) → MeOH (maceration, r.t., successively)	CC NP Silica gel (gr. Petrol-EtOAc-MeOH)	TLC NP Silica gel (CHCl_3_-MeOH)	TLC NP Silica gel (CHCl_3_)	[20]
	**52**, **54**, **56**	Light petrol → Et_2_O → MeOH (Soxhlet, successively)	CC NP Silica gel (gr. *n*-Hexane-Et_2_O-MeOH)	CC NP Silica gel (gr. *n*-Hexane-Acetone)	HPLC NP Silica gel (CHCl_3_-MeOH) → PLC NP Silica gel (CHCl_3_-Acetone)	[24]
	**53–56**	Light petrol → Et_2_O → MeOH (Soxhlet, successively)	CC NP Silica gel (CHCl_3_-EtOAc)	PLC NP Silica gel (Benzene-EtOAc) → PLC NP Silica gel (CHCl_3_-Acetone) or PLC NP Silica gel (Benzene-EtOAc) → crystallization (MeOH)		[26]
	**57**	MeOH (reflux)	CC RP Diaion HP20 (gr. MeOH-H_2_O → MeOH → EtOH → EtOAc)	CC NP Silica gel (gr CHCl_3_-MeOH-H_2_O)	CC NP Silica gel (CHCl_3_-MeOH-H_2_O) → CC RP C_18_ (MeOH-H_2_O)	[13]
	**49**, **51**, **52**, **59**	MeOH (r.t.)	LLE H_2_O-EtOAc	CC NP Silica gel (gr. *n*-Hexane-Acetone)	CC RP C_18_ (gr. MeOH-H_2_O) → HPLC RP C_18_ (MeOH-H_2_O) → crystallization (*n*-Hexane-Acetone) or CC RP C_18_ (gr. MeOH-H_2_O) → HPLC RP C_18_ (MeOH-H_2_O) → HPLC NP Silica gel (CHCl_3_-MeOH)	[27]
	**53**	Petrol → Et_2_O (Soxhlet, successively)	CC NP Silica gel (gr. n-Hexane-Et_2_O)	CC NP Silica gel (gr. CHCl_3_-EtOAc)	CC NP Silica gel (Benzene-EtOAc) → TLC NP Silica gel (*n*-Hexane-Et_2_O-dioxane)	[25]
	**49–52**	Petrol → Et_2_O → MeOH (Soxhlet, successively)	CC NP Silica gel (gr. CHCl_3_-MeOH)	TLC NP Silica gel (Benzene-EtOAc)	PLC NP Silica gel (Benzene-EtOAc)	[14]

* The fraction obtained with the solvent highlighted in underlined font was used in further isolation steps; r.t. = room temperature; ** extraction solvent not disclosed; DCM = CH_2_Cl_2_; LLE = liquid–liquid extraction; CC = column chromatography; TLC = thin-layer chromatography; PLC = preparative thin-layer chromatography; HPLC = high-performance liquid chromatography; MPLC = medium-pressure liquid chromatography; GPC = gel permeation chromatography; CPC = centrifugal partition chromatography; NP = normal phase; RP = reversed phase; FA = formic acid; TFA = trifluoroacetic acid.

## Data Availability

Not applicable.

## Supplementary Materials

The following supporting information can be downloaded at: https://www.mdpi.com/article/10.3390/molecules28145420/s1, Table S1: Biological activity of spiro-flavonoids with information on the models used and results for the substances tested.

## Abbreviations

12-HETE	12-Hydroxy-5,8,10,14-eicosatetraenoic acid
AbsC	Absolute configuration
ABTS	2,2′-Azino-bis(3-ethylbenzothiozoline-6-sulfonic acid) diammonium salt
AChE	Acetylcholinesterase
BChE	Butyrylcholinesterase
CC	Column chromatography
CDA	Chiral derivatizing agent
CHS	Chalcone synthase
COSY	Correlation spectroscopy
COX	Cyclooxygenase
DEPT	Distortion enhancement by polarization transfer
DPPH	2,2-Diphenyl-1-picrylhydrazyl
EC_50_	Half maximal effective concentration
ECD	Electronic circular dichroism
GIAO	Gauge-independent atomic orbital
GPC	Gel permeation chromatography
HHT	12-Hydroxy-5,8,10-heptadecatrienoic acid
HIV-1	Human immunodeficiency virus type 1
HMBC	Heteronuclear multiple bond correlation
IC_50_	Half maximal inhibitory concentration
iNOS	Inducible nitric oxide synthase
KS	Kaposi’s sarcoma
LLE	Liquid–liquid extraction
LOX	Lipoxygenase
LPS	Lipopolysaccharide
LTB_4_	Leukotriene B_4_
MAPK	Mitogen-activated protein kinases
MIC	Minimum inhibitory concentration
MPLC	Medium-pressure liquid chromatography
MTPA	α-methoxy-α-trifluoromethylphenylacetic acid
MTT	3-(4,5-Dimethylthiazol-2-yl)-2,5-diphenyl-2*H*-tetrazolium bromide
NF-κB	Nuclear factor kappa-light-chain-enhancer of activated B cells
NMR	Nuclear magnetic resonance
NO	Nitric oxide
NOESY	Nuclear Overhauser effect spectroscopy
NOR 1	(±)-(*E*)-Methyl-2-[(*E*)-hydroxyimino]-5-nitro-6-methoxy-hex-3-enamide
NP	Normal phase
PAF	Platelet-activating factor
PARP-1	Poly(ADP-ribose) polymerase 1
PGE_2_	Prostaglandin E_2_
PLC	Preparative thin-layer chromatography
PTP1B	Human protein tyrosine phosphatase 1B
QM	Quantum mechanical
RelC	Relative configuration
ROS	Reactive oxygen species
RP	Reversed phase
STS	Stilbene synthase
TDDFT	Time-Dependent Density-Functional Theory
TEAC	Trolox Equivalent Antioxidant Capacity
THMS	*trans*-3,3′,5,5′-Tetrahydroxy-4′-methoxystilbene
TLC	Thin-layer chromatography
TNF-α	Tumor necrosis factor-α
UV/Vis	Ultraviolet/Visible
VCD	Vibrational circular dichroism
VEGF	Vascular endothelial growth factor
XRD	X-ray diffraction
