# Supplementary material for: Spiro-Flavonoids in Nature: A Critical Review of Structural Diversity and Bioactivity
Source: Molecules. 2023 Jul 14;28(14):5420. doi: 10.3390/molecules28145420 (PMC10385819; doi:10.3390/molecules28145420)
Supplement: Supplementary file 1 [file molecules-28-05420-s001.zip › molecules-2480595-supplementary.pdf]

molecules

Supplementary Materials

Spiro-Flavonoids in Nature: A Critical Review of Structural Diversity and Bioactivity

Łukasz Pecio [1,2], Solomiia Pecio [1,*], Tomasz Mroczek [2] and Wiesław Oleszek [1]

[1] Department of Biochemistry and Crop Quality, Institute of Soil Science and Plant Cultivation—State Research Institute, 8 Czartoryskich Street, 24-100 Puławy, Poland; lpecio@iung.pulawy.pl (Ł.P.); wieslaw.oleszek@iung.pulawy.pl (W.O.)
[2] Department of Chemistry of Natural Products, Medical University of Lublin, 1 Chodźki Street, 20-093 Lublin, Poland; tmroczek@pharmacognosy.org
* Correspondence: skozachok@iung.pulawy.pl

Abstract: Based on literature data from 1973 to 2022, this work summarizes reports on spiro-flavonoids with a spiro-carbon at the center of their structure and how this affects their isolation methods, stereochemistry, and biological activity. The review collects 65 unique structures, including spiro-biflavonoids, spiro-triflavonoids, spiro-tetraflavonoids, spiro-flavostilbenoids, and scillascillin-type homoisoflavonoids. Scillascillin-type homoisoflavonoids comprise spiro[bicyclo[4.2.0]octane-7,3′-chromane]-1(6),2,4-trien-4′-one, while the other spiro-flavonoids contain either 2*H*,2′*H*-3,3′-spirobi[benzofuran]-2-one or 2′*H*,3*H*-2,3′-spirobi[benzofuran]-3-one in the core of their structures. Spiro-flavonoids have been described in more than 40 species of eight families, including Asparagaceae, Cistaceae, Cupressaceae, Fabaceae, Pentaphylacaceae, Pinaceae, Thymelaeaceae, and Vitaceae. The possible biosynthetic pathways for each group of spiro-flavonoids are summarized in detail. Anti-inflammatory and anticancer activities are the most important biological activities of spiro-flavonoids, both in vitro and in vivo. Our work identifies the most promising natural sources, the existing challenges in assigning the stereochemistry of these compounds, and future research perspectives.

Keywords: spiro-flavonoids; spiro-biflavonoids; spiro-triflavonoids; spiro-tetraflavonoids; spiro-flavostilbenoids; scillascillin-type homoisoflavonoids; stereochemistry; biosynthesis; biological activity; isolation

Table S1. Biological activity of spiro-flavonoids.

Activity	Model	Assay	Effect of Reference Standard	Spiro-Flavonoid Name	Effect of Tested Comp.	[1] Ref.
Antioxidant	Cell-free	DPPH	IC_{50} (ascorbic acid) = 3.50±0.02 μg/mL	Larixinol (**1**)	IC_{50}=30.54±0.29 μg/mL	[1]
	Cell-free	ABTS	[2] TEAC (quercetin) = 2.6±0.02	Larixinol	TEAC =1.79±0.02	[2]
				Yuccaone A (**29**)	TEAC =1.04 ± 0.02	[2]
				Yuccaol A (**36**)	TEAC =0.96 ± 0.04	[2]
				Yuccaol B (**37**)	TEAC = 1.09 ± 0.08	[2]
				Yuccaol C (**38**)	TEAC = 1.6 ± 0.01	[2]
				Yuccaol D (**39**)	TEAC = 1.42 ± 0.02	[2]
				Yuccaol E (**40**)	TEAC = 1.85 ± 0.13	[2]
				Gloriosaol A (**44**)	TEAC = 5.55 ± 0.07	[3]
				Gloriosaol B (**45**)	TEAC = 3.00 ± 0.08	[3]
				Gloriosaol C (**46**)	TEAC = 5.6 ± 0.01, the highest activity	[3]
				Mix of gloriosaols D (**47**) &E (**48**)	TEAC = 4.91 ± 0.10	[3]
	Cell-free	β-carotene/linoleic acid autoxidation	[3] AA_{60} ([5] BHT) = 71.8<br>[4] AA_{120} (BHT) = 61.2	Larixinol	AA_{60} = 24.4; [4] AA_{120} = 51.0	[2]
				Yuccaone A	AA_{60} = 40.6; AA_{120} = 43.4	[2]
				Yuccaol A	AA_{60} = 52.6; AA_{120} = 72.1	[2]
				Yuccaol B	AA_{60} = 76.3; AA_{120} = 72.1	[2]
				Yuccaol C	AA_{60} = 59.5; AA_{120} = 71.7	[2]
				Yuccaol D	AA_{60} = 66.4; AA_{120} = 66.2	[2]
				Yuccaol E	AA_{60} = 74.3; AA_{120} = 79.3	[2]
	Cell-free	[6] 15-LOX inhibition	EC_{50} (ascorbic acid) = 21.52 μg/mL	Yuccaol B	EC_{50} = 9.66 μg/mL	[4]
				Gloriosaol A	EC_{50} = 12.34 μg/mL	[4]
	Cell-free	DPPH, H_2O_2 scavenging, NO scavenging	DPPH, [7] RSA (Trolox) = 92.1%	Scillascillin (**49**)	RSA (DPPH) = 6.3%; SA (H_2O_2) = 40.6%; SA (NO) = 37% at 500 μM	[5]
			DPPH, RSA (curcumin) = 94.1%	2-Hydroxy-scillascillin (**51**)	RSA (DPPH) = 33.6%; SA (H_2O_2) = 33.4%; SA (NO) = 25.1% at 500 μM	[5]

			DPPH, RSA (α-tocopherol) = 88.1%<br>H_2O_2, [8] SA (Trolox) = 100 %<br>NO, SA (Trolox) = 86%	Isomuscomosin (**52**)	RSA (DPPH) = 91.7%; EC_{50} = 22.9 μM; SA (H_2O_2) = 99.3%; SA (NO) = 14.3% at 500 μM	[5]
				Scillavone A (**59**)	RSA (DPPH) = 38.3%; SA (H_2O_2) = 85.4%; SA (NO) = 26.2% at 500 μM	[5]
**Anti-inflammatory**	Cell line RAW 264.7	NO release (Griess reagent) activated with [9] LPS	[10] IR (aminoguanidine) = 51% at C = 50 μg/mL	Larixinol (**1**)	IR (%) = 75% at 100 μg/mL, IC_{50} = 60.0 μg/mL	[6]
				3-*Epi*-larixinol (**2**)	Not active	[6]
				3,2′-*Epi*-larixinol (**3**)	Not active	[6]
	Cell line RAW 264.7	NO release (Griess reagent) activated with LPS	IR (aminoguanidine) = 50% at 25 μM	Daphnodorin C (**22**)	Not active	[7,8]
				Daphnodorin I (**24**)	Not active	[7,8]
				2″-Methoxy-daphnodorin C (**26**)	IR = 32% at C=100 μg/mL	[7,8]
				2″-Methoxy-2-*epi*-daphnodorin C (**27**)	IR = 58% at C=100 μg/mL	[7,8]
	Cell line NCI-H292	[11] PMA-stimulated, Western blot (antibodies against p-IκB, IκB, p-NF-κB, p-CREB, p-c-jun, p-ERK1/2, ERK1/2, p-JNK, JNK, p-p38, p38)	—	Daphnodorin C (**22**)	Suppressed NF-κB and [12] MAP kinase signaling pathways (JNK and p38).<br>↓ TNF-α secretion with IC_{50} = 19.26 μM, and ↓ [13] MUC5AC protein release with IC_{50} = 21.42 μM,	[9]
				Daphnodorin I (**24**)	Not active	[9]
	Mice – 6-old male C57BL/6	[14] COPD using cigarette smoke- and LPS-exposed	Roflumilast at a dose of 5 mg/kg. Its efficacy comparable to Daphnodorin C at 20 mg/kg	Daphnodorin C (**22**)	↓ TNF-α, ↓ IL-6, ↓ [15] ROS, ↓ elastase activity in [16] BALF from COPD mice at doses of 10 and 20 mg/kg.	[9]
	Cell-based [17] PGHS-1, [18] PGHS-2 [19] PMNLs	COX-1, COX-2, [20] PGE_2 concentration (EIA kit), [21] LTB_4 formation mediated by 5- LOX (EIA kit)	COX-1, IC_{50} (indomethacin) = 0.9 μM.	Yuccaol A (**36**)	IC_{50} (COX-1) = 12.5 μM, IC_{50} (COX-2) = 75.5 μM<br>IC_{50} (LTB_4) >125 μM	[10]
			COX-2, IC_{50} (NS-398) = 2.6 μM.	Yuccaol B (**37**)	IC_{50} (COX-1) = 10.3 μM, IC_{50} (COX-2) = 71.5 μM<br>IC_{50} (LTB_4) >125 μM	[10]

			LTB_4, IC_{50} (zileuton) = 5.0 μM	Yuccaol C (**38**)	IC_{50} (COX-1) = 33.8 μM, IC_{50} (COX-2) >125 μM<br>IC_{50} (LTB_4) >125 μM	[10]
				Yuccaol D (**39**)	IC_{50} (COX-1) = 87.7 μM, IC_{50} (COX-2) >125 μM<br>IC_{50} (LTB_4) >125 μM	[10]
				Yuccaol E (**40**)	IC_{50} (COX-1) = 14.2 μM, IC_{50} (COX-2) = 65.9 μM<br>IC_{50} (LTB_4) >125 μM	[10]
	Cell line J774.A1	NO (Griess reagent), Western blot (antibody for [22] iNOS), [23] NF-κB ([24] EMSA) release induced with LPS	—	Yuccaol A (**36**)	IR (NO) = 6.7 % at 0.1 μM<br>IR (NO) = 11.8 % at 1 μM<br>IR (NO) = 19.7 % at 10 μM<br>IR (NO) = 55.8 % at 100 μM<br>iNOS, NF-kB – not active	[11]
				Yuccaol B (**37**)	**Not active**	[11]
				Yuccaol C (**38**)	IR (NO) = 8.6 % at 0.1 μM<br>IR (NO) = 24.7 % at 1 μM<br>IR (NO) = 36.8 % at 10 μM<br>IR (NO) = 87.5 % at 100 μM<br>IR (iNOS) = 46.2 % at 0.1 μM<br>IR (iNOS) = 52 % at 1 μM<br>IR (iNOS) = 56.6 % at 10 μM<br>IR (iNOS) = 81.7 % at 100μM<br>↓ NF-κB binding activity	[11]
	Cell line RAW 264.7	iNOS, IL-1β, and IL-6 mRNA levels activated with LPS ([25] RT-qPCR)	—	Yuccaol C (**38**)	↓ iNOS, ↓ IL-1β, ↓ IL-6 mRNA level at 100 μM	[12]
				Yuccaol D (**39**)	↓ IL-1β, ↓ IL-6 mRNA level at 100 μM	[12]
				Yuccaol E (**40**)	↓ iNOS, ↓ IL-1β, ↓ IL-6 mRNA level at 100 μM	[12]
				Yuccalide A (**41**)	**Not active**	[12]
				Yuccalide B (**42**)	↓ iNOS mRNA level at 100 μM	[12]
				Yuccalide C (**43**)	**Not active**	[12]

	Cell based C0858	COX-2, measured by the conversion of PGG2 to PGH2 (Cayman Fluorescence activity kit)	[26] EA (DuP-607) = 47.0 ± 15.5% at 304 nM	2-Hydroxy-7-*O*-methyl-scillascillin (**50**)	EA (2*R* **50**) = 66.7 ± 17.5%, EA (2*S* **50**) = 89.0±29.2% at 10 μM	[13]
				Socialinone (**63**)	EA = 101.1±32.6% at 10 μM.	[13]
	Cell free<br>Cell free<br>Cell line RAW 264.7	LOX (decoloration of methyleneblue)<br>Hyaluronidase inhibition (Morgan–Elson method)<br>NO (Griess reagent) generation induced with LPS	LOX, IC_{50} (nordihydroguaiaretic acid) = 9.61 μM<br>[27] HI, IC_{50} (tannic acid) < 550 μM	Scillascillin (**49**)	IC_{50} (LOX) < 1000 μM<br>IC_{50} ([27]HI) < 1000 μM<br>↓ NO production at 50 μM	[14]
				2-Hydroxy-scillascillin (**51**)	IC_{50} (LOX) = 788 μM<br>IC_{50} ([27]HI) < 1000 μM<br>↓ NO production at 50 μM	[14]
				Isomuscomosin (**52**)	IC_{50} (LOX) = 927 μM<br>IC_{50} ([27]HI) < 1000 μM<br>↓ NO production at 50 μM	[14]
				Scillavone A (**59**)	IC_{50} (LOX) < 1000 μM<br>IC_{50} ([27]HI) < 1000 μM<br>↓ NO production at 10 and 50 μM	[14]
	Cell based cell	COX-1,<br>COX-2 measured, cell microsomal fractions<br>PGE_2 concentration	[28]$IPGE_2MC$ (indomethacin) = 70-80% at 5 μM<br>Inhibition COX-1 (indomethacin) = 60-70% at 12.5 μM<br>Inhibition COX-2 (indomethacin) = 60-70% at 200 μM	Scillascillin (**49**)	Inhibition PGE_2 in microsomal cells = 34 ± 1.7 % at 250 μg/mL<br>COX-1, COX-2 **not active**	[15]
				Isomuscomosin (**52**)	$IPGE_2MC$ = 65 ± 4.7% at 250 μg/mL<br>COX-1, COX-2 **not active**	[15]
				3′,5-Dihydroxy-4′,7- dimethoxyspiro[2*H*-1-benzopyran-3(*4H*),7′-bicyclo[4.2.0]octa[1,3,5]-trien]-4-one (**56**)	$IPGE_2MC$ = 47 ± 1.2% at 250 μg/mL<br>COX-1, COX-2 **not active**	[15]
	Cell line J774.1	NO (Griess reagent), PGE_2 conc. (PGE_2 correlate-EIA kit), TNF-α, IL-6, COX-2, and iNOS mRNA levels (RT-qPCR) induced with LPS	—	Protosappanin D (**65**)	IC_{50} (NO) = 9.6 μM<br>IC_{50} (PGE_2) = 7.8 μM<br>IC_{50} (TNF-α) = 14.2 μM<br>IC_{50} (IL-6) = 3.0 μM<br>IC_{50} (COX-2) = 21.4 μM<br>IC_{50} (iNOS) = 13.2 μM	[16]
	Cell-free			Yuccalechin B (**6**)	IC_{50} (AChE) = 294.18±5.26 μM	[17]

					BChE – **not active**	
				Yuccalechin C (**7**)	IC_{50} (AChE) = 655.18 ±6.35 μM<br>BChE – **Not active**	[17]
				Yuuccaol A (**36**)	IC_{50} (AChE) = 267.1 ± 1.4 μM<br>IC_{50} (BChE) = 235.3 ± 4.6 μM	[18]
				Yuccaol B (**37**)	IC_{50} (AChE) = 43.3 ± 2.7 μM<br>IC_{50} (BChE) = 81.3 ± 2.5 μM	[18]
				Yuccaol C (**38**)	IC_{50} (AChE) = 169.3 ± 2.4 μM<br>IC_{50} (BChE) = 229.7 ± 3.7 μM	[18]
**Neuroprotective**		AChE (from electric eel), BChE (from horse serum) inhibition (modified Ellman method)	AChE, IC_{50} (galantamine) = 2.3±0.3 μM<br>BChE, IC_{50} (galantamine) = 124.0±4.1 μM	Yuccaol D (**39**)	IC_{50} (AChE) = 442.7 ± 6.2 μM<br>IC_{50} (BChE) = 685.4 ± 6.1 μM	[18]
				Yuccaol E (**40**)	IC_{50} (AChE) = 173.3 ± 2.7 μM<br>IC_{50} (BChE) = 148.1 ± 5.8 μM	[18]
				Yuccalide A (**41**)	IC_{50} (AChE) = 428.2 ± 5.6 μM<br>BChE – **not active**	[18]
				Gloriosaol A (**44**)	IC_{50} (AChE) = 45.4 ± 2.4 μM<br>IC_{50} (BChE) = 64.9 ± 3.3 μM	[18]
				Gloriosaol C (**46**)	IC_{50} (AChE) = 271.5 ± 3.8 μM<br>IC_{50} (BChE) = 218.6 ± 4.6 μM	[18]
				Gloriosaol D (**47**)	IC_{50} (AChE) = 230.7 ± 5.1 μM<br>IC_{50} (BChE) = 909.4 ± 6.1 μM	[18]
				Gloriosaol E (**48**)	IC_{50} (AChE) = 95.0 ± 3.6 μM<br>IC_{50} (BChE) = 155.7 ± 4.4 μM	[18]
	Adult zebrafish	Y-maze, Novel tank diving scopolamine-induced model (recorded the swimming behavior)	—	Yuccaol B (**6**)<br>Gloriosaol A (**44**)	Attenuated the Sco-induced amnesia and anxiety, and improved precognitive and anxiolytic activities, to the level of the control group (untreated with Sco)	[4]<br>[4]
**Anticancer and antitumor**	*Chang* liver cells	[29] NOR 1 (transformed cells induced by NO activation, were observed under light microscope (×100))	[30] IR (curcumin) = 2.1 at 350 nmol	Abiesinol A (**12**)	IR = 2.1 at 350 nmol	[19]
				Abiesinol B (**13**)	IR = 2.1 at 350 nmol	[19]
				Abiesinol C (**14**)	IR = 2.1 at 350 nmol	[19]
				Abiesinol D (**15**)	IR = 2.1 at 350 nmol	[19]
				Abiesinol E (**1**)	IR = 2.0 at 350 nmol	[19]

			Abiesinol F (**4**)	IR = 2.0 at 350 nmol	[19]
Mice female ICR (6 weeks)	Skin carcinogenesis induced by ([31] PN; $ONOO^-$ — a single dose and [32] TPA — 2 times a week for 20 weeks)	—	Abiesinol A (**12**), administrated orally 0.0025% of the body weight for 2 weeks before TPA application	At the end of the experiments, the average number of papillomas per mouse was decreased by 2 compared to the negative control.	[19]
Cell line PANC-1	Cytotoxicity (counting Kit-8)	—	3-*Epi*-larixinol (**2**)<br>Fragranol B (**8**)<br>Fragranol C (**9**)<br>Fragranol A (**30**)	Not active at 100 μM<br>Not active at 100 μM<br>Not active at 100 μM<br>Not active at 100 μM	[20,21]<br>[21]<br>[21]<br>[20]
Cell linesA549, MCF-7, HeLa, BEAS-2B	Cytotoxicity (MTT)	Doxorubicin, IC_{50} (HeLa) = 1.41±0.35 μg/mL<br>IC_{50} (MCF-7) = 1.52±0.57 μg/mL<br>IC_{50} (A549) = 1.29±0.27 μg/mL<br>IC_{50} (BEAS-2B) = 0.09±0.01 μg/mL	Genkwanol A (**23**)	Cell viability inhibition (A549) = 16.8 % at 20 μg/mL<br><br>MCF-7, HeLa, BEAS-2B — Not active	[22]
Cell based	Cytotoxicity (microtubule polymerization)	IC_{50} (colchicine) = 10.2 ± 0.6 μM<br>IC_{50} (vinblastine) = 2.4 ± 0.1 μM	Genkwanol A (**23**)	IC_{50} = 112 ± 4 μM	[23]
Cell based Hsp90	Affinity toward Hsp90 protein (surface plasmon resonance analyses)	Radicicol at 0.025–1 mM	Genkwanol A (**23**)<br>Daphnodorin I (**24**)<br>4′-Methylgenkwanol A (**25**)<br>2′′-Hydroxygenkwanol A (**28**)	[33] K_D = 7.8±1.1 μM at 0.025–1 mM<br>K_D = 1.6±0.4 μM at 0.025–1 mM<br>K_D = 1.5±0.8 μM at 0.025–1 mM<br>K_D = 0.5±0.1 μM	[24]<br>[24]<br>[24]<br>[24]

				at 0.025–1 mM, the best stability of the spiro-biflavonoid/Hsp90 complex	
Kaposi's sarcoma (KS) cells	Mitogenic (XTT colorimetric assay and Coulter counter), Western blot (*p*38 and *p*42/44, protein content measured by Lowry method) induced with [34] VEGF. Migration (image analysis) activated by [35] PAF. Enzyme [36] AT, [37] TA_L activities (liquid scintillation counting) induced with VEGF	—	Yuccaol A (**36**)	↓ KS cell proliferation, ↓ MAP kinase signaling pathways (p38 and p42/44), ↓ AT, ↑ TA_L, ↓ (from 7.6 to 6.1 μm/h) KS cell motility activated by PAF, at 25 μM	[25]
			Yuccaol B (**37**)	↓ KS cell proliferation, ↓ MAP kinase signaling pathways (p38 and p42/44), ↓ AT, ↑ TA_L, ↓ (from 7.6 to 5.6 μm/h) KS cell motility activated by PAF, at 25 μM	[25]
			Yuccaol C (**38**)	↓↓ KS cell proliferation, ↓ MAP kinase signaling pathways (p38 and p42/44), ↓ AT, ↑ TA_L, ↓↓ (from 7.6 to 5.3 μm/h) KS cell motility activated by PAF, at 25 μM	[25]
Cell lines MCF7, HepG2, U937, Molt4, Jurkat	Cell proliferation and viability (acid phosphatase and tetrazolium salt-based methods, cytometric counts). Cell necrosis ([38] PI staining). Apoptosis (PI incorporation in permeabilized cells, DNA conc. was measured by flow cytometry) induced with etoposide. Mitochondria depolarization ([39] TMRE). Western blot (cytochrome *c*). Intracellular	—	Gloriosaol A (**44**)	EC_{50} (U937) = 16.3±1.07 μM<br>EC_{50} (MOLT4) = 22.6±2.62 μM<br>EC_{50} (Jurkat T) = 29.5±3.01 μM<br>EC_{50} (MCF7) = 34.2±3.49 μM<br>EC_{50} (HepG2) = 43.6±5.05 μM	[26]
			Gloriosaol B (**45**)	EC_{50} (U937) = 29.5±2.86 μM<br>EC_{50} (MOLT4) = 31.8±3.47 μM<br>EC_{50} (Jurkat T) = 37.5±4.50 μM<br>EC_{50} (MCF7) = 40.1±3.93 μM<br>EC_{50} (HepG2) = 59.3±6.17 μM	[26]
			Gloriosaol C (**46**)	EC_{50} (U937) = 8.04±0.56 μM<br>EC_{50} (MOLT4) = 12.1±1.27 μM<br>EC_{50} (Jurkat T) = 17.4±2.09 μM<br>EC_{50} (MCF7) = 22.0±2.62 μM<br>EC_{50} (HepG2) = 30.4±3.65 μM	[26]

		redox state (exposed to oxidant [40] *t*-BOOH, measuring [41] DCF fluorescence)			Induced apoptosis at 10-25 μM in cell line U937 and switched to necrosis at doses above 30 μM. Caused mitochondrial depolarisation and cytochrome *c* release at doses > 10 μM in U937 cells. Showed the best pro-oxidant and antioxidant effects in U937 cells.	
	Cell lines HREC, HUVEC, ARPE-19, 92-1, Y79	Cell proliferation (alamarBlue based fluorescence methodology)	—	Scillascillin (**49**)<br>Isomuscomosin (**52**)<br>Muscomosin (**53**)<br>Socialinone (**63**)<br>5-Hydroxy-2′,3′,4′,7-tetrametoxyspiro[4*H*-1-benzopyran-3(2*H*),7′-bicyclo[4.2.0]octa[1,3,5]-trien]-4-one (**64**)	Not active<br>Not active<br>Not active<br>Not active<br>Not active	[27]<br>[27]<br>[27]<br>[27]<br>[27]
	Cell lines HL-60, TIG-3	MTT	Etoposide, IC_{50} (HL-60) = 0.3±0.01 μM, IC_{50} (TIG-3) = 11.2±1.4 μM. Cisplatin, IC_{50} (HL-60) = 1.6±0.05 μM, IC_{50} (TIG-3) = 4.5±0.1 μM.	5,7,5′-Trihydroxy-4′-methoxy-6-methylspiro[2*H*-1-benzopyran-3(4*H*),7′-bicyclo[4.2.0]octa[1,3,5]-trien]-4-one (**57**)	IC_{50} (HL-60) > 40 μM<br>IC_{50} (TIG-3) > 40 μM	[28]
	Cell lines MCF-7, DU-145	MTT	Doxorubicin, IC_{50} (MCF-7) = 1.86±0.003 μg/mL, IC_{50} (DU-145) = 13.71±0.02 μg/mL	Scillascillin (**49**)	IC_{50} (MCF-7) = 9.59±0.01 μg/mL<br>IC_{50} (DU-145) = 11.32±0.04 μg/mL	[29]
**Cytotoxicity**	Cell line RAW 264.7	MTT	—	Larixinol (**1**)	No cytotoxicity at a concentration to 100 μg/mL	[6]
	Cell line NCI-H292	Cell Counting Kit-8	—	Daphnodorin I (**24**)<br>Daphnodorin C (**22**)	No cytotoxicity at 2.5—20 μM<br>No cytotoxicity at 2.5—20 μM	[9]<br>[9]
	Cell line J774.A1	MTT	—	Yuccaol A (**36**)	No cytotoxicity at 0.1— 100 μg/mL	[11]

				Yuccaol B (**37**)		[11]
				Yuccaol C (**38**)		[11]
	Salmonella typhimurium strains TA97, TA98, TA100 and TA102	*Salmonella*/mammalian microsome mutagenicity test (Ames test)	—	Yuccaol A (**36**)	Non-toxic and non-mutagenic at 10 to 500 µg/plate	[30]
				Yuccaol B (**37**)	Non-toxic and non-mutagenic at 10 to 500 µg/plate	[30]
				Yuccaol C (**38**)	Non-toxic and non-mutagenic at 10 to 500 µg/plate	[30]
**Antibacterial, antifungal and antiviral**	*Pyricularia oryzae*	Antifungal (suspensions of spores and mycelia were inoculated onto leaves of host-plant – *Oriza sativa)*.	—	Daphnodorin C (**22**)	Protective value = 89-90 % at 200-500 ppm	[31]
	Pyricularia oryzae; cell line CEM	Antifungal; antiviral (HIV-1, XTT formazan)	[42] MMDC (griseofulvin) = 50.4±2.7 µM, MMDC (nocodazole) = 50.2±3.2 µM MMDC (fursarielin A) = 15.3±0.9 µM; EC_{50} (AZT) = 0.186 µM	Genkwanol A (**23**)	MMDC = 45.8±0.5 µM EC_{50} = 4.00 µM	[23]
	MT-4 cells, MOLT-4 (clone 8)	Antiviral (HIV-1 (IIIB), XTT method). Anti-HIV in [42] PBL (p24 capture enzyme-linked immunosorbent assay). Reverse transcriptase. HIV-1-infected cell fusion (XTT, counted under a microscope)	[43] DDC, EC_{50} (HIV-1 cytopatbogenicity in MT-4 cells) = 0.013±0.009 µg/mL; IC_{50} = 38 ± 7 µg/mL. EC_{50} (HIV-1 p24 antigen production in PBL) = 0.00033 µg/mL, IC_{50} = 43±2 µg/mL.	Daphnodorin C (**22**)	HIV-1 cytopatbogenicity in MT-4 cells: EC_{50} = 3.6±0.5 µg/mL; IC_{50} = 38 ± 1 µg/mL<br><br>HIV-1 p24 antigen production in PBL cells: EC_{50} = 1.9±1 µg/mL, IC_{50} = 65±6 µg/mL<br>weak inhibitory effects on the reverse transcriptase of H1V-1	[32]
				Scalliscillin (**49**)	MIC = 0.5 mM	[33]

Staphylococcus aureus	Antibacterial (bioautographic, microplate assays)	MIC (neomycin) = 0.0025 mM	Isomuscomosin (**52**)	MIC = 3.95 mM<br>BC = 1.97 mM	[33]
			3′,5 -Dihydroxy-4′7′-dimethoxyspiro[2*H*-1-benzopyran-3(4*H*),7′-bicyclo[4.2.0]octa[1,3,5]-trien]-4-one (**56**)	MIC = 7.60 mM	[33]

[1] Ref. — References. [2] TAEC — Trolox Equivalent Antioxidant Capacity value. [3] AA_{60} — Antioxidant activity value after 60 min. [4] AA_{120} — Antioxidant activity value after 120 min. [5] BHT — 2,6-di-tert-butyl-4-methoxyphenol. [6] 15-LOX — 15-lipoxygenase. [7] RSA – Radical scavenging activity. [8] SA – Scavenging activity. [9] LPS – lipopolysaccharide. [10] IR — Inhibition rate (%). [11] PMA – phorbol 12-myristate 13-acetate. 12 MAP – Mitogen-activated protein. [13] MUC5AC – mucin 5AC (it is a major mucin protein secreted from the airway surface epithelium). [14] COPD —chronic obstructive pulmonary disease. [15] ROS – Reactive oxygen species. [16] BALF – bronchoalveolar lavage fluid. [17] PGHS-1 – Prostaglandin H synthase obtained from ram seminal vesicles for COX-1 assay. [18] PGHS-2 – obtained from sheep placental cotyledons for COX-2 assay. [19] PMNLs — Polymorphonuclear leukocytes were isolated from venous human blood for LTB4 formation assay. [20] PGE2 — Prostaglandin E2. [21] LTB4 — Leukotriene B4. [22] iNOS — Inducible nitric oxide synthase. [23] NF-κB — nuclear factor kappa-light-chain-enhancer of activated B cells. [24] EMSA — Electrophoretic mobility shift assay. [25] RT-qPCR — Reverse transcription quantitative PCR. [26] EA – Enzyme activity. [27] HI – Hyaluronidase inhibition (%). [28] IPGE2MC – Inhibition PGE2 in microsomal cells (%).[29] NOR 1 – (±)-(E)-Methyl-2-[(E)-hydroxyimino]-5-nitro-6-methoxy- hex-3-enamide. [30] IR – Inhibitory ration. [31] PN – peroxynitrite. [32] TPA – 12-*O*-tetradecanoylphorbol-13-acetate. [33] K_D – Thermodynamic constants measured by SPR for the interaction between tested compounds and immobilized Hsp90. [34] VEGF – Vascular endothelial growth factor. [35] PAF – Platelet activating factor. [36] AT – Acetyl-CoA:ly.so-PAF acetyltransferase. [37] TA_L – Lysophospholipids. [38] PI – Propidium iodide. [39] TMRE – Tetramethylrhoda- mine ethyl ester. [40] *t*-BOOH – *tert*-Butylhydroperoxide. [41] DCF – Dichlorofluorescein. [42] MMDC –Morphological deformation concentration. [42] PBL – Peripheral blood lymphocytes. [43] DDC – 2′,3′-Dideoxycytidine- 5′-triphosphate.

References

1. Baldan, V.; Sut, S.; Faggian, M.; Dalla Gassa, E.; Ferrari, S.; De Nadai, G.; Francescato, S.; Baratto, G.; Dall'Acqua, S. *Larix Decidua* Bark as a Source of Phytoconstituents: An LC-MS Study. *Molecules* **2017**, *22*, 1974, doi:10.3390/molecules22111974.
2. Piacente, S.; Montoro, P.; Oleszek, W.; Pizza, C. *Yucca Schidigera* Bark: Phenolic Constituents and Antioxidant Activity. *J. Nat. Prod.* **2004**, *67*, 882–885, doi:10.1021/np030369c.
3. Bassarello, C.; Bifulco, G.; Montoro, P.; Skhirtladze, A.; Benidze, M.; Kemertelidze, E.; Pizza, C.; Piacente, S. *Yucca Gloriosa*: A Source of Phenolic Derivatives with Strong Antioxidant Activity. *J. Agric. Food Chem.* **2007**, *55*, 6636–6642, doi:10.1021/jf071131n.
4. Pecio, Ł.; Kozachok, S.; Brinza, I.; Stefan Boiangiu, R.; Hritcu, L.; Mircea, C.; Flavia Burlec, A.; Cioanca, O.; Hancianu, M.; Wronikowska-Denysiuk, O.; et al. Neuroprotective Effect of *Yucca Schidigera* Roezl Ex Ortgies Bark Phenolic Fractions, Yuccaol B and Gloriosaol A on Scopolamine-Induced Memory Deficits in Zebrafish. *Molecules* **2022**, *27*, 3692, doi:10.3390/molecules27123692.
5. Nishida, Y.; Wada, K.; Toyohisa, D.; Tanaka, T.; Ono, M.; Yasuda, S. Homoisoflavones as the Antioxidants Responsible from Bulbs of *Scilla Scilloides*. *Nat. Prod. Res.* **2013**, *27*, 2360–2362, doi:10.1080/14786419.2013.830218.
6. Li, Y.-L.; Yang, X.-W.; Li, S.-M.; Tang, J.; Tian, J.-M.; Peng, X.-Y.; Huang, D.-S.; Zhang, W.-D. Two New Spirobiflavonoids from *Abies Chensiensis* with Moderate NO Production Inhibitory Activity. *Planta Med.* **2009**, *75*, 1534–1537, doi:10.1055/s-0029-1185746.
7. Liang, S.; Tian, J.-M.; Feng, Y.; Liu, X.-H.; Xiong, Z.; Zhang, W.-D. Flavonoids from *Daphne Aurantiaca* and Their Inhibitory Activities against Nitric Oxide Production. *Chem. Pharm. Bull.* **2011**, *59*, 653–656, doi:10.1248/cpb.59.653.
8. Liang, S.; Tang, J.; Shen, Y.-H.; Jin, H.-Z.; Tian, J.-M.; Wu, Z.-J.; Zhang, W.-D.; Yan, S.-K. Biflavonoids from *Daphne Feddei* and Their Inhibitory Activities against Nitric Oxide Production. *Chem. Pharm. Bull.* **2008**, *56*, 1729–1731, doi:10.1248/cpb.56.1729.
9. Ryu, H.W.; Lee, J.W.; Kim, M.O.; Lee, R.W.; Kang, M.J.; Kim, S.M.; Min, J.H.; Oh, E.S.; Song, Y.N.; Jung, S.; et al. Daphnodorin C Isolated from the Stems of *Daphne Kiusiana* Miquel Attenuates Airway Inflammation in a Mouse Model of Chronic Obstructive Pulmonary Disease. *Phytomedicine* **2022**, *96*, 1–12, doi:10.1016/j.phymed.2021.153848.
10. Wenzig, E.M.; Oleszek, W.; Stochmal, A.; Kunert, O.; Bauer, R. Influence of Phenolic Constituents from *Yucca Schidigera* Bark on Arachidonate Metabolism *in Vitro*. *J. Agric. Food Chem.* **2008**, *56*, 8885–8890, doi:10.1021/jf801289m.
11. Marzocco, S.; Piacente, S.; Pizza, C.; Oleszek, W.; Stochmal, A.; Pinto, A.; Sorrentino, R.; Autore, G. Inhibition of Inducible Nitric Oxide Synthase Expression by Yuccaol C from *Yucca Schidigera* Roezl. *Life Sci.* **2004**, *75*, 1491–1501, doi:10.1016/j.lfs.2004.03.013.
12. Nakashima, K.; Abe, N.; Oyama, M.; Inoue, M. Yuccalides A-C, Three New Phenolic Compounds with Spiro-Structures from the Roots of *Yucca Gloriosa*. *Fitoterapia* **2016**, *111*, 154–159, doi:10.1016/j.fitote.2016.04.009.
13. Waller, C.P.; Thumser, A.E.; Langat, M.K.; Crouch, N.R.; Mulholland, D.A. COX-2 Inhibitory Activity of Homoisoflavanones and Xanthones from the Bulbs of the Southern African *Ledebouria Socialis* and *Ledebouria Ovatifolia* (Hyacinthaceae: Hyacinthoideae). *Phytochemistry* **2013**, *95*, 284–290, doi:10.1016/j.phytochem.2013.06.024.
14. Nishida, Y.; Sugahara, S.; Wada, K.; Toyohisa, D.; Tanaka, T.; Ono, M.; Yasuda, S. Inhibitory Effects of the Ethyl Acetate Extract from Bulbs of Scilla Scilloides on Lipoxygenase and Hyaluronidase Activities. *Pharm. Biol.* **2014**, *52*, 1351–1357, doi:10.3109/13880209.2014.891140.
15. Du Toit, K.; Elgorashi, E.E.; Malan, S.F.; Drewes, S.E.; Van Staden, J.; Crouch, N.R.; Mulholland, D.A. Anti-Inflammatory Activity and QSAR Studies of Compounds Isolated from Hyacinthaceae Species and Tachiadenus Longiflorus Griseb. (Gentianaceae). *Bioorganic Med. Chem.* **2005**, *13*, 2561–2568, doi:10.1016/j.bmc.2005.01.036.
16. Washiyama, M.; Sasaki, Y.; Hosokawa, T.; Nagumo, S. Anti-Inflammatory Constituents of Sappan Lignum. *Biol. Pharm. Bull.* **2009**, *32*, 941–944, doi:10.1248/bpb.32.941.
17. Pecio, Ł.; Alilou, M.; Kozachok, S.; Orhan, I.E.; Eren, G.; Deniz, F.S.S.; Stuppner, H.; Oleszek, W. Yuccalechins A-C from the *Yucca Schidigera* Roezl Ex Ortgies Bark: Elucidation of the Relative and Absolute Configurations of Three New Spirobiflavonoids and Their Cholinesterase Inhibitory Activities. *Molecules* **2019**, *24*, doi:10.3390/molecules24224162.
18. Pecio, Ł.; Alilou, M.; Kozachok, S.; Orhan, I.E.; Eren, G.; Şenol Deniz, F.S.; Stuppner, H.; Oleszek, W. Absolute Configuration of Spiro-Flavostilbenoids from *Yucca Schidigera* Roezl Ex Ortgies: First Indication of (2*R*)-Naringenin as the Key Building Block. *Phytochemistry* **2023**, *207*, 113584, doi:10.1016/j.phytochem.2022.113584.
19. Wada, S.-I.; Hitomi, T.; Tokuda, H.; Tanaka, R. Anti-Tumor-Initiating Effects of Spiro-Biflavonoids from *Abies Sachalinensis*. *Chem. Biodivers.* **2010**, *7*, 2303–2308, doi:10.1002/cbdv.201000147.
20. Omar, A.M.; Sun, S.; Kim, M.J.; Tawila, A.M.; Dibwe, D.F.; Phrutivorapongkul, A.; Toyooka, N.; Awale, S. Fragranol A: A New Class of Spiro-Triflavanoid Hybrid with an Unprecedented Carbon Skeleton from *Anneslea Fragrans*. *Tetrahedron Lett.* **2020**, *61*, 152099, doi:10.1016/j.tetlet.2020.152099.
21. Omar, A.M.; Sun, S.; Kim, M.J.; Tawila, A.M.; Dibwe, D.F.; Phrutivorapongkul, A.; Toyooka, N.; Awale, S. Highly Oxygenated Spiro-Biflavanoids from *Anneslea Fragrans* Twigs. *Phytochem. Lett.* **2020**, *40*, 21–25, doi:10.1016/j.phytol.2020.09.005.
22. Emerce, E.; Gürbüz, P.; Doğan, Ş.D.; Kadioglu, E.; Süntar, I. Cytotoxic Activity-Guided Isolation Studies on *Fumana Procumbens* (Dunal) Gren. & Godr. *Rec. Nat. Prod.* **2019**, *13*, 189–198, doi:10.25135/rnp.98.18.07.331.
23. Hu, K.; Kobayashi, H.; Dong, A.; Iwasaki, S.; Yao, X. Antifungal, Antimitotic and Anti-HIV-1 Agents from the Roots of Wikstroemia Indica. *Planta Med.* **2000**, *66*, 564–567, doi:10.1055/s-2000-8601.
24. Malafronte, N.; Vassallo, A.; Dal Piaz, F.; Bader, A.; Braca, A.; De Tommasi, N. Biflavonoids from *Daphne Linearifolia* Hart. *Phytochem. Lett.* **2012**, *5*, 621–625, doi:10.1016/j.phytol.2012.06.008.

25. Balestrieri, C.; Felice, F.; Piacente, S.; Pizza, C.; Montoro, P.; Oleszek, W.; Visciano, V.; Balestrieri, M.L. Relative Effects of Phenolic Constituents from *Yucca Schidigera* Roezl. Bark on Kaposi's Sarcoma Cell Proliferation, Migration, and PAF Synthesis. *Biochem. Pharmacol.* **2006**, *71*, 1479–1487, doi:10.1016/j.bcp.2006.01.021.
26. Nigro, P.; Bloise, E.; Turco, M.C.; Skhirtladze, A.; Montoro, P.; Pizza, C.; Piacente, S.; Belisario, M.A. Antiproliferative and Pro-Apoptotic Activity of Novel Phenolic Derivatives of Resveratrol. *Life Sci.* **2007**, *81*, 873–883, doi:10.1016/j.lfs.2007.07.010.
27. Schwikkard, S.; Whitmore, H.; Sishtla, K.; Sulaiman, R.S.; Shetty, T.; Basavarajappa, H.D.; Waller, C.; Alqahtani, A.; Frankemoelle, L.; Chapman, A.; et al. The Antiangiogenic Activity of Naturally Occurring and Synthetic Homoisoflavonoids from the Hyacinthaceae (Sensu APGII). *J. Nat. Prod.* **2019**, *82*, 1227–1239, doi:10.1021/acs.jnatprod.8b00989.
28. Matsuo, Y.; Kurihara, R.; Akagi, N.; Mimaki, Y. Two New Homoisoflavonoids from the Bulbs of *Bessera Elegans*. *Nat. Prod. Commun.* **2014**, *9*, 1725–1727, doi:10.1177/1934578X1400901216.
29. Chinthala, Y.; Chinde, S.; Kumar, A.N.; Srinivas, K.V.N.S.; Kumar, J.K.; Sastry, K.P.; Grover, P.; Ramana, M.V. Anticancer Active Homoisoflavone from the Underground Bulbs of *Ledebouria Hyderabadensis*. *Pharmacognosy Res.* **2014**, *6*, 303–305, doi:10.4103/0974-8490.138272.
30. Czeczot, H.; Podsiad, M.; Skrzycki, M.; Stochmal, A.; Oleszek, W. Evaluation of the Mutagenic Activity of Phenolics from the Bark of *Yucca Schidigera* Roezl. *Acta Pol. Pharm.* **2003**, *60*, 357–362.
31. INAMORI, Y.; TAKEUCHI, K.; BABA, K.; KOZAWA, M. Antifungal and Insecticidal Activities of Daphnodorins A, B and C. *Chem. Pharm. Bull.* **1987**, *35*, 3931–3934, doi:10.1248/cpb.35.3931.
32. Yusa, K.; Oh-hara, T.; Tsukahara, S.; Baba, K.; Taniguchi, M.; Kozawa, M.; Takeuchi, S.; Hara, H.; Tsuruo, T. Inhibition of Human Immunodeficiency Virus Type 1 (HIV-1) Replication by Daphnodorins. *Antiviral Res.* **1994**, *25*, 57–66, doi:10.1016/0166-3542(94)90093-0.
33. Du Toit, K.; Elgorashi, E.E.; Malan, S.F.; Mulholland, D.A.; Drewes, S.E.; Van Staden, J. Antibacterial Activity and QSAR of Homoisoflavanones Isolated from Six Hyacinthaceae Species. *South African J. Bot.* **2007**, *73*, 236–241, doi:10.1016/j.sajb.2007.01.002.

Disclaimer/Publisher's Note: The statements, opinions and data contained in all publications are solely those of the individual author(s) and contributor(s) and not of MDPI and/or the editor(s). MDPI and/or the editor(s) disclaim responsibility for any injury to people or property resulting from any ideas, methods, instructions or products referred to in the content.
